# An underwater vest containing an antioxidant MXene hydrogel for sensitive recognition of fish locomotion

**DOI:** 10.1038/s41378-024-00675-8

**Published:** 2024-03-22

**Authors:** Chengxiu Yang, Jiafei Hu, Lihui Liu, Shaowei Wu, Mengchun Pan, Yan Liu, Haomiao Wang, Peisen Li, Qi Zhang, Weicheng Qiu, Huihui Luo

**Affiliations:** College of Intelligence Science and Technology, National University of Defense Technology, Changsha, 410073 China

**Keywords:** Carbon nanotubes and fullerenes, Electrical and electronic engineering, Sensors

## Abstract

The perception of fish locomotion is important for understanding their adaptive behaviors and ethological characteristics. However, the main strategy used for extracting fish attitudes involves the use of a vision-based monitoring system, which is limited in its range of observation and cannot perform tracking for long times. Here, we report the use of a wearable tagging electronic device, referred to as an underwater vest, to capture the surrounding flow field disturbances triggered by swimming or momentary postural changes. All of these goals were achieved by integrating a pair of pseudocapacitive pressure-sensing units and a flexible circuit board. Notably, additional conditions, such as variable hydraulic pressures and minimal changes in fish posture, require high stability and sensitivity of the sensing units. Thus, hybrid hydrogel electrodes were developed through cross-linking MXene with holey-reduced graphene oxide nanosheets and further modification with 1-ethyl-3-methylimidazolium dicyanamide ionic liquids, which increased the interfacial capacitance and long-term interfacial activity of the MXene. Consequently, the sensing unit exhibited ultrahigh sensitivity (*S*_max_~136,207 kPa^−1^) in an aquatic environment for 60 days and superior high-pressure resolution (10 Pa) within a wide working range of 1 MPa. Ultimately, an underwater vest integrated with such sensing units clearly distinguished and recorded fish locomotion. We believe that the designed device may open avenues in flow field monitoring and ocean current detection and provide new insights into the development of sensitive underwater tagging.

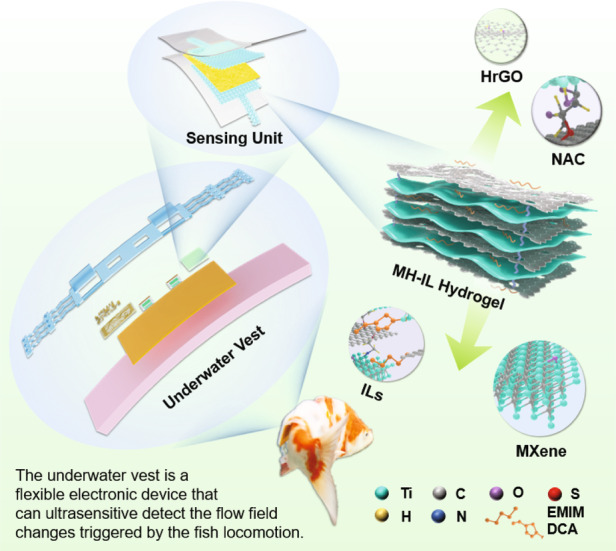

## Introduction

Over the past century, anthropogenic factors have exerted great influence on rivers, lakes, and oceans. Overfishing, installation of underwater artificial equipment, discharges, and leaking pollutants destroy the habitats of fish, which profoundly affects their adaptive behaviors, such as feeding, reproduction, and migration. Most basic behaviors of fish can be identified by their specific locomotor postures^1^. Consequently, observation of the typical details of locomotion is essential for understanding fish behaviors and speculating on environmental variables. The mainstream method for determining fish locomotion involves the use of vision-based monitoring systems, such as underwater cameras and fish sounders^2^. Since visible light is inevitably attenuated when it travels underwater, fixed-position visual monitoring systems cannot perform tracking in natural aquatic environments for long times^3^. Given these limitations, recently developed wearable electronic devices have received widespread attention because of their noninvasive methods of attachment and conformal structures, which do not disturb natural fish motion; additionally, these devices provide long-term and animal-comfortable alternatives for monitoring fish locomotion and collecting physiological information^4,5^. Specifically, changes in the movement postures of fish, such as sudden turning, accelerating, and sinking, can trigger disturbances in the hydrodynamic pressure field^6^. Therefore, these flexible and wearable devices attached to fish can be used to perceive the pressure variations around them and infer their ethological characteristics^7,8^.

Nevertheless, emerging traceable monitoring has not been as completely implemented in fish locomotion recognition as we hoped. First, in comparison with exposure to an atmospheric environment, the additional hydraulic pressures that arise because fish primarily inhabit underwater areas at certain depths may hinder the sensitivity and resolution of measuring small disturbances^9,10^. It remains challenging to maintain the same high performance of the device seen in terrestrial environments over a wide pressure range. Second, fish with slow-swimming movements exhibit minimal changes in posture, which hardly disturbs the surrounding flow field; to this end, only highly sensitive pressure sensors can accurately recognize such situations under high pressure. Thus, it is particularly important to improve the sensitivity for observing every move of fish with additional waterproof packaging. Another practical issue arises from coupling factors, such as undercurrents and ocean currents, which can result in additional disturbances in the flow field and cause misjudgment of fish locomotion. Therefore, it is necessary to design a highly sensitive wearable device that can withstand water pressure at great depths for long periods of time and exclude the influence of disturbing flow fields.

The pseudocapacitance-pressure-sensing mechanism involves an active interfacial reversible redox reaction with a unit area capacitance (UAC) of up to hundreds of µF/cm^2^ under external mechanical loading; this mechanism has the highest capacitive sensitivity among all pressure sensors^11–13^. Recently, we reported a pseudocapacitance-based artificial hair cell—a biomimetic underwater pressure sensor with modified MXene (Ti_3_C_2_T_x_) electrodes. Notably, the artificial hair cells are capable of recognizing weak underwater waves with sensitivities as high as tens of thousands of kPa^−1^, which are at least three to four orders of magnitude greater than those of existing parallel plate capacitive sensors^14^. Therefore, artificial hair cells mimic the sensitive unit of the lateral line on fish and are able to detect small fluctuations in the flow field triggered by mechanical stimuli such as falling objects and swinging of the fishtail. However, two critical characteristics of MXene materials are detrimental to maintaining long-term high interfacial activity underwater: self-restacking and oxidative degradation^15,16^. Specifically, for MXene nanosheets, 2D materials with abundant functional groups are prone to stacking because of the van der Waals forces between them, and this is followed by the loss of many active surface sites in contact with the dielectric layer^17^. Another essential problem stems from the fact that MXene nanosheets are likely to react with oxidizing electrolytes (such as sulfuric acid and phosphoric acid) and water molecules that infiltrate the interior of the sensor^18–20^, gradually forming TiO_2_ nanoparticles from their edge to the surface and seriously reducing the interfacial capacitance^21^.

By utilizing the MXene-based ultrahigh interfacial pseudocapacitance effect, we first reported a hydrogelation strategy that enabled MXenes to maintain high underwater activity for long times. Specifically, holey-reduced graphene oxide (HrGO) was cross-linked with MXene and *N*-acetyl-L-cysteine (NAC) and subsequently modified with 1-ethyl-3-methylimidazolium dicyanamide (EMIM DCA) as an ionic liquid. The synergistic modification increased the interlayer spacing of the MXenes, suppressed self-stacking, and established a defensive layer on the surface, which improved the antioxidant properties of the materials. The proposed MXene/HrGO-ionic liquid hydrogel (named MH(α)-IL), where α denotes the weight of HrGO in the hydrogel, as shown in Table S1), is an active electrode material with a highly resilient double-network acidic hydrogel dielectric layer integrated into an ultrasensitive pressure-sensing unit. This material has a maximum sensitivity of up to 136,207 kPa^−1^ in the aquatic environment for a 60-day period and exhibits superior underwater pressure resolution with a wide working range of 1 MPa. Notably, considering the tricky challenges of fish locomotion recognition, we prepared a wearable and water-stable underwater vest with a pair of symmetrically arranged blind holes embedded with sensing units that monitor normal pressure fluctuations to the left and right sides triggered by fish locomotion. As illustrated by the experimental results, the designed underwater vest clearly distinguishes the surrounding flow field perturbations formed as fish free swim, especially for transient locomotion changes, such as turning, sinking, and surfacing.

## Results

### Preparation and characterization of the MH-IL hydrogel

**Fig. 1:**
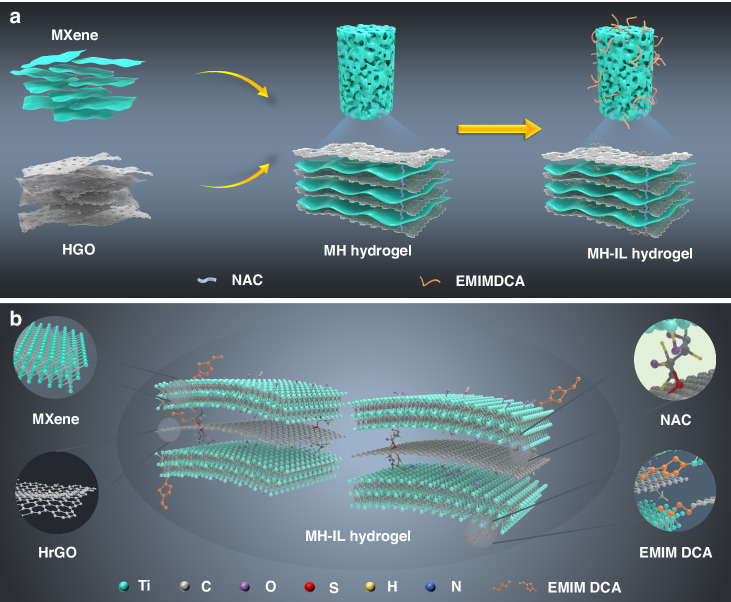
**Synthesis of the MH-IL hydrogel.** **a** Schematic showing the preparation of the MH-IL hydrogel. **b** The molecular interactions between the MXene, HrGO, NAC, and EMIM DCA

Figure 1 illustrates the preparation process of the MH-IL hydrogel and the intrinsic mechanisms for NAC cross-linking and EMIM DCA modification (the molecular structure is shown in Fig. S1, Supplementary Information). The Ti_3_C_2_T_x_ nanosheets were fabricated through etching of the Al layers in the precursor MAX phase (Ti_3_AlC_2_) with a LiF/HCl mixture^22^. As an intercalated structure between neighboring MXene nanosheets, the holey graphene oxide (HGO) exhibited multiple nanopores on its entire basal surface, which provided adequate nanochannels for ion transport (Fig. S2, Supplementary Information). The MXene colloidal suspension with the Tyndall effect (Fig. S3, Supplementary Information) was easily and uniformly mixed with the HGO aqueous dispersion, in which the electrostatic interactions assisted the assembly of the MXene and HGO layers^23^. NAC, which served as a bridge across the HGO and MXene nanosheets through terminal amino and sulfhydryl functional groups, was added to the aforementioned mixture as a cross-linking agent to enable polymerization into an MH hydrogel (Fig. S4, Supplementary Information). Subsequently, the interfacial effect occurred when EMIM DCA dropped into the MH hydrogel precursor^24^, enabling EMIM DCA to adsorb and modify the surface of the MXene via the ionic-electronic coupling mechanism. Finally, the MH-IL hydrogel was prepared via natural precipitation (Fig. S5, Supplementary Information).

**Fig. 2:**
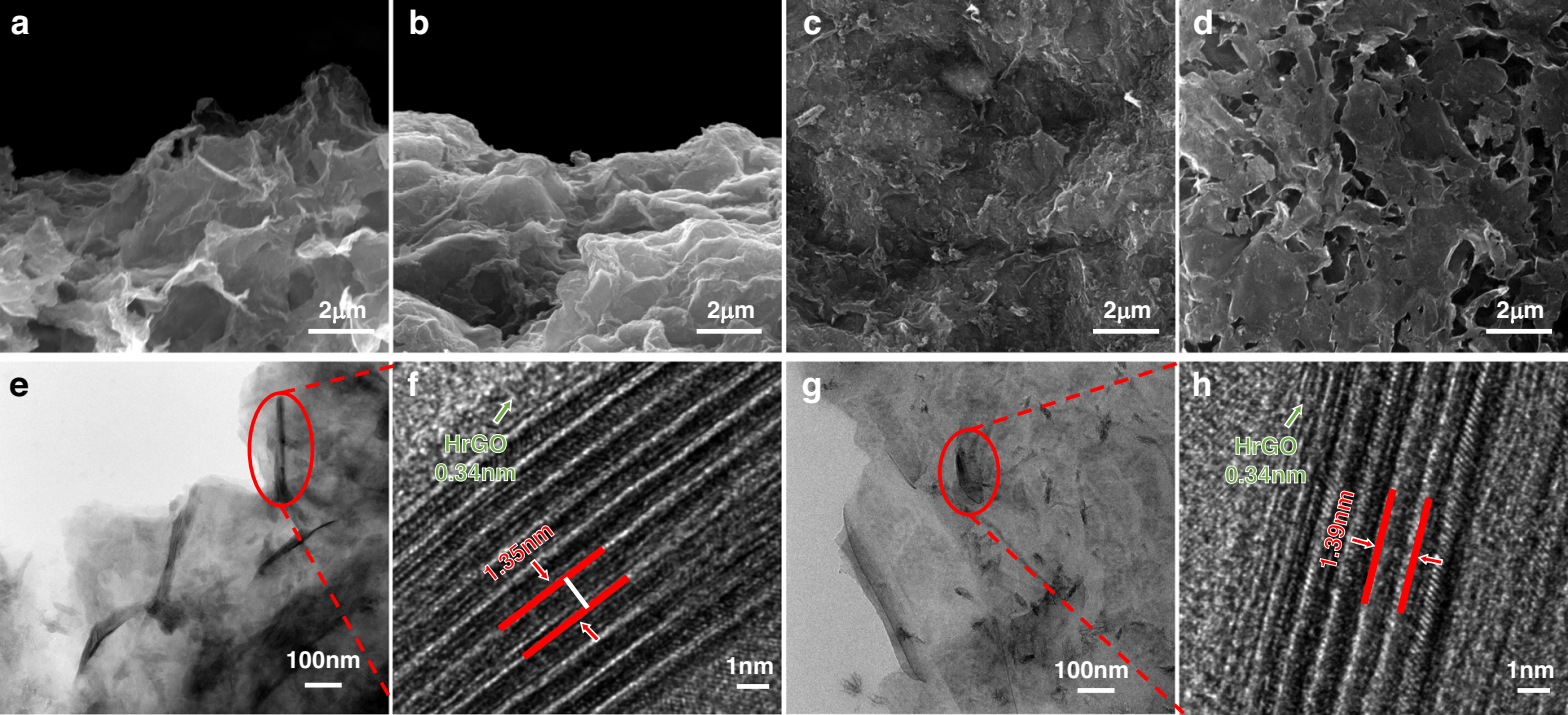
**Morphology of the MH-IL hydrogel.** **a**, **b** Cross-sectional SEM images of MH(30) and MH(30)-IL hydrogel-derived films, respectively. **c**, **d** Top-view SEM images of MH(30) and MH(30)-IL hydrogel-derived films, respectively. **e**, **f** TEM and HRTEM images of MH (30), respectively. **g**, **h** TEM and HRTEM images of MH (30)-IL, respectively

To observe the effects of NAC and EMIM DCA on the morphology and characteristics of the original MXene film, MH(30) and MH(30)-IL hydrogel-derived films were prepared by vacuum-assisted filtration (Fig. S6, Supplementary Information). The compact hierarchical and well-defined restacked structure of the pure MXene film was verified from the cross-sectional view of the scanning electron microscopy (SEM) image (Fig. S7a, Supplementary Information). In comparison, the SEM image of the MH(30) hydrogel-derived film from the same perspective showed a porous, interconnected 3D network with pore walls composed of intercross-linked MXene and HrGO nanosheets, as shown in Fig. 2a. Notably, Fig. 2b demonstrates that the MH(30)-IL hydrogel-derived film had a 2D lamellar internal structure, which was apparently different from those of the films made without modification by EMIM DCA. The differences in microstructure may be due to the charged EMIM DCA molecules embedded between the intrinsic nanosheets, which provided stronger electrostatic interactions and led to a denser network structure^20^. According to the top-view SEM images of the MH(30) and MH(30)-IL samples (Fig. 2c, d, respectively), the surface features of the hybrids showed rougher surfaces with more uneven bulges or depressions than the pure MXene film (Fig. S7b, Supplementary Information), possibly originating from the significant differences in size between the Ti_3_C_2_T_x_ and HrGO nanosheets^25^, as well as changes in the interlayer spacing after assembling HrGO and the corresponding cross-linking agent and ionic liquids. The transmission electron microscopy (TEM) image of the MH(30) sample revealed ultrathin MXene lamellae with some layered wrinkles, whose interiors consisted of assembled MXene and HrGO nanosheets (Fig. 2e). High-resolution transmission electron microscopy (HRTEM) was used to display the lattice planes of these wrinkles (Fig. 2f). For instance, the lattice spacing of partial parallel fringes was approximately 0.34 nm, corresponding to the lamellar distance of graphene^26^. Moreover, a much larger interplanar spacing of 1.35 nm, greater than that of pure MXene reported previously^27^, was clearly observed, indicating that surface-to-surface stacking between HrGO and MXene had occurred. It is apparent from the TEM image of the MH(30)-IL sample that the modified MXene lamellae exhibited decreases in transparency and the number of wrinkles, along with an abundance of irregular particulate matter on the surface (Fig. 2g). As shown in Fig. 2h, upon intercalation of the ionic liquid into the hybrid mixture layer, the lattice spacing increased to 1.39 nm.

**Fig. 3:**
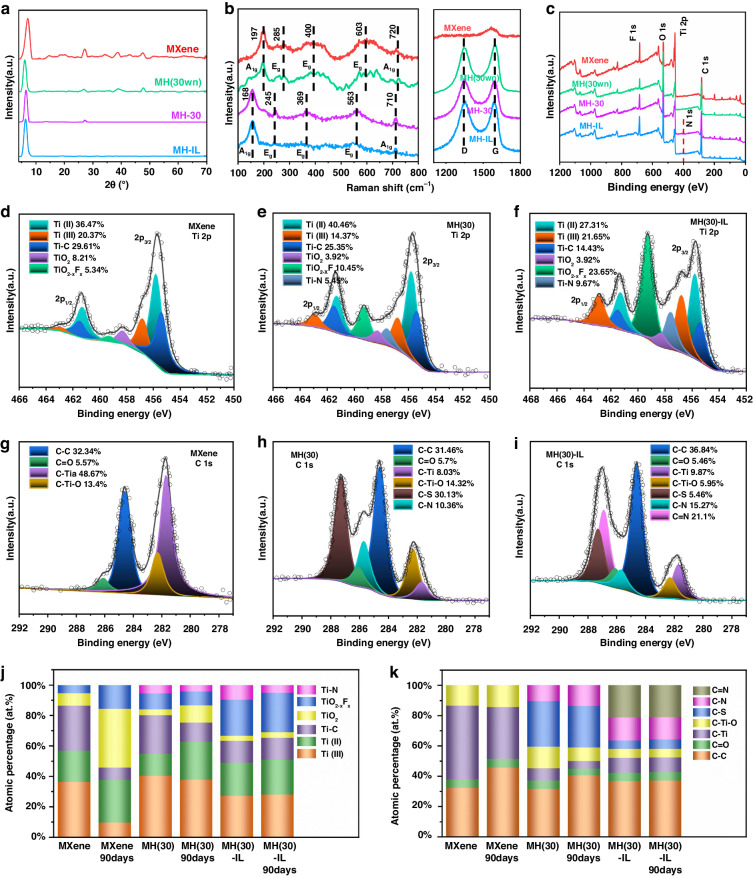
**Characteristics of the MXene, MH (30wn), MH (30), and MH (30)-IL.** **a** XRD patterns, **b** Raman spectra, and **c** XPS survey spectra for pure MXene, MH (30wn), MH (30), and MH (30)-IL, respectively. Ti 2p XPS spectra for **d** MXene, **e** MH(30), and **f** MH(30)-IL. C 1 s XPS spectra for **g** MXene, **h** MH(30), and **i** MH(30)-IL. Histograms showing the atomic percentages with different aging times for the Ti 2p **j** and C 1 s **k** regions

We used X-ray diffraction (XRD) and Raman spectroscopy to investigate the crystalline phases and chemical compositions of the MXene before and after doping with HGO, NAC, and EMIM DCA. Figure 3a displays the XRD patterns of the obtained MXene, MH(30), MH(30) without NAC (MH(30wn)) and MH(30)-IL. The (002) peak for MXene was located at 7.185° due to a lattice spacing of ~1.22 nm. The MXene nanosheets with negatively charged edges interacted with HGO, which has a positively charged surface, via electrostatic interactions. The diffraction peak for the hybrid shifted to 6.238°, indicating an increase in the lattice spacing to ~1.41 nm. Apparently, the peak shifted to 6.544°, and the lattice spacing decreased to 1.35 nm after cross-linking by NAC, demonstrating that the cross-linking process resulted in a tighter assembly than that from electrostatic interactions, perhaps via hydrogen bonding and van der Waals interactions. After modification with EMIM DCA, several EMIM^+^ ions were inserted between the MH(30) hybrid lamellae through Coulombic interactions, which caused a slight increase in the lattice spacing to approximately 1.39 nm^23^, which was consistent with the aforementioned HRTEM results. Furthermore, the introduction of HGO and HrGO caused a decrease in the stacking order of the MXene lamellae, as evidenced by the decreased intensity of the (002) peak, resulting in a decrease in the conductivity and density of the hybrid material. However, the incorporation of EMIM DCA, which has a high ionic conductivity, increased the peak intensity. Interestingly, both pure MXene and MH(30wn) exhibited distinct peaks at 2θ values of 27.6°, 37°, and 48°, which were attributed to anatase titanium dioxide^28^. While a weak peak at 2θ = 27.6° was still present for MH(30), MH(30)-IL showed none of these diffraction peaks, indicating that the ionic liquid prevented oxidation of the Ti on the MXene flakes. To investigate the presence of Ti atoms and verify the crystal structure after a period of degradation, the XRD patterns determined for samples stored for 90 days are displayed in Fig. S8 (Supplementary Information). Irreversible degradation of the MXene nanosheets was indicated by a substantial weakening of the (002) peaks for both MXene and MH(30wn), accompanied by a noticeable increase in the intensity of the characteristic peak for anatase titanium dioxide. Nevertheless, the absence of TiO_2_-related peaks in MH(30)-IL provided evidence that the internal crystal structure of MXene remained unaltered. Furthermore, the electrical conductivities of the specimens, which reflected the degree of oxidation over time, were evaluated experimentally. After standing for 90 days in the air, the conductivity of the MXene sample decreased from 62.1 to 12.4 S cm^−1^, a decrease of 80.03% compared with those of the cross-linked MH(30) (46.79%) and the MH(30)-IL (22.8%) doped with ionic liquids (Fig. S9, Supplementary Information).

Figure 3b shows the Raman spectra of four obtained samples, i.e., MXene, MH(30wn), MH(30), and MH(30)-IL. The obtained MXene and MH(30wn) samples exhibited similar vibrational peaks below 800 cm^−1^, since the *E*_g_ in-plane vibrations of C, Ti, and other atoms in the lamellar surface terminal groups exhibited peaks at 285, 400, and 603 cm^−1^, respectively, and the *A*_1g_ out-of-plane vibrations at 197 and 720 cm^−1^ were contributed to Ti and C atoms. Remarkably, the *E*_g_ and *A*_1g_ vibration peaks of MH(30) and MH(30)-IL were seen at lower wavenumbers than those of MXene and MH(30wn), which may be due to an increase in the ratio of edge-to-basal-planes of the modified nanosheets and therefore an increase in the concentration of defects^29^. After introducing the HGO and HrGO nanosheets, intense peaks were observed at 1348 and 1599 cm^−1^, corresponding to the D and G bands of graphene, respectively. In particular, the intensity ratio of the D to G bands increased from 0.95 (MH(30wn)) to 1.06 (MH(30)) and 1.03 (MH(30)-IL) due to the reduction capacity of NAC and the resulting enhanced edge effect^30^.

X-ray photoelectron spectroscopy (XPS) was also used to analyze the effects of cross-linking and oxidation resistance after modification. Figure 3c indicates that F, O, C, and Ti were found in all samples, whereas MH(30) and MH(30)-IL exhibit N 1 s peaks at ~402 eV. The Ti 2p spectrum of pure MXene was deconvoluted into Ti 2p_1/2_ and 2p_3/2_ peaks for Ti-C (461.5 and 455.4 eV), Ti(II) (461.3 and 455.8 eV), Ti(III) (462.9 and 456.8 eV), TiO_2_ (458.3 eV), TiO_2-x_F_x_ (459.3 eV) (Fig. 3d), and Ti-N (457.6 eV) in the MH (30) and MH(30)-IL after NAC introduction. To verify the effects of the antioxidants in NAC and EMIM DCA, the Ti 2p spectra were deconvoluted, as shown in Fig. 3e, f, respectively. The TiO_2_ component decreased from 8.21% in the pure MXene to 3.92% in MH(30) and 3.29% in MH(30)-IL, and the Ti-C component decreased from 29.61% in pure MXene to 25.35% in MH(30) and 14.43% in MH(30)-IL, indicating that the amounts of TiO_2_ and amorphous carbon formed on the surface of Ti_3_C_2_T_x_ after oxidation were lower in the presence of NAC and EMIM DCA. Notably, after being immersed in water for 90 days, the proportion of TiO_2_ in the pure MXene increased sharply to 38.46%, while the proportion of Ti-C decreased to 8.1%. However, in the case of MH(30)-IL, the two important oxidative indicators remained almost unchanged, indicating that the addition of NAC and EMIM DCA ensured the underwater stability of the sample. (Fig. 3j and Fig. S10a-c, Supplementary Information). The C 1 s peak of MXene was attributed to C-C (284.6 eV), C=O (286.1 eV), C-Ti (281.7 eV), and C-TiO (282.3 eV) species (Fig. 3g). Since NAC was cross-linked between the HGO and MXene sheets, the C-N (285.7 eV) and C-S (287.3 eV) peaks appeared in the spectra of the MH(30) and MH(30)-IL samples (Fig. 3h, i). Notably, MH(30)-IL showed a new peak corresponding to C=N (286.9 eV) in the C 1 s spectrum, which was unique to EMIM DCA and implied that it has a modified MH(30). By comparing the three samples after 90 days of storage in water, we found that the C-C bond component of the MXene had increased significantly, but that of MH(30)-IL hardly changed, probably due to the increased amorphous carbon proportion (Fig. 3k and Fig. S10d–f, Supplementary Information). Subsequently, O 1 s spectra were used to verify the influence of these proposed antioxidant measures on the Ti-OH (532 eV) and TiO-Ti (529.4 eV) spectra (Fig. S11, Supplementary Information). This was because Ti-OH had the lowest work function and, thus, the highest reactivity among the surface functional groups of the MXenes^31^. The oxidation of Ti_3_C_2_T_x_ started from numerous reactions triggered at the surface-exposed Ti-OH sites, which reacted with the incoming -OHs through hydrogen bonding interactions^32^. According to density functional theory (DFT) calculations, the Ti-OH groups on the surface of Ti_3_C_2_T_x_ were dehydrated, and an additional O layer was formed above the Ti layer, resulting in TiO_2_ particles^33^. The intensity of the Ti-OH peak in the MXene spectrum decreased from 53.47% to 43.91% (MH (30)) and 42.93% (MH (30)-IL) after cross-linking by NAC and further modification with EMIM DCA, respectively. This indicated that some of the Ti-OH functional groups were substituted by NAC and eliminated by EMIM DCA through hydrogen annealing^34^. These reactions altered the surface chemistry in a synergetic way, leading to enhanced oxidation resistance of the MXenes.

These findings revealed the causes of the cross-linking and chemical stabilities of the materials: (i) Ti-N bonds were formed through dehydration reactions between the Ti-OH species on the surface of the MXene and the -NH_2_ groups at the end of NAC, while the oxygen-containing functional groups on HGO were bound to the -SH groups on NAC. As a result, NAC served as a cross-linker between the MXene and HGO lamellae; (ii) after the cross-linking reaction and IL treatment, a substantial decrease in the number of active Ti-OH groups occurred sequentially, resulting in a decrease in the intrinsic activity of the MXene and passivation; and (iii) a defensive layer was constructed on the surface of the MXene through strong electrostatic interactions and Ti-N_x_ interactions between imidazole ILs, which hindered the approach of the surrounding -OH radicals (Fig. S1, Supplementary Information).

### Design and characterization of ultrasensitive pressure-sensing units

**Fig. 4:**
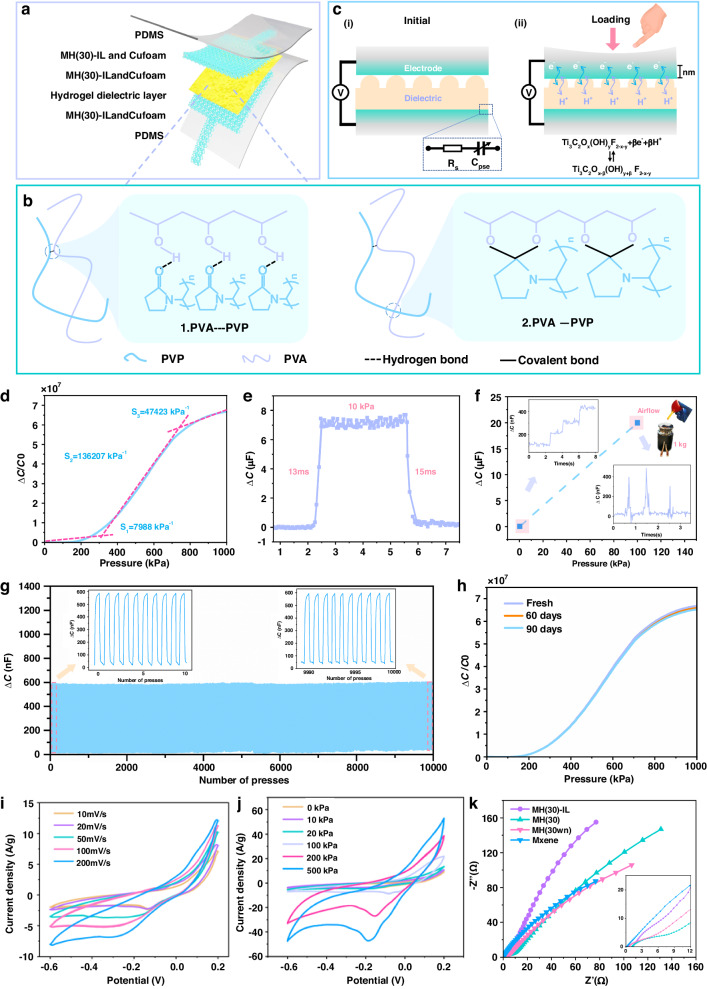
**Structure, sensing mechanisms, sensing performances, and characterization of the pressure-sensing unit.** **a** Schematic illustration of the sensing unit. **b** Molecular illustration of the interaction between PVA and PVP. **c** Sensing mechanisms of the unit under applied and removed loads. **d** Capacitance-pressure response curve of the sensing unit with MH(30)-IL electrodes. **e** The response and reset times of the sensing unit. **f** Pressure resolution tests loaded at base pressures of 0 kPa and 100 kPa. **g** Compression-release stability over 10,000 cycles. **h** Capacitance-pressure response curve of the unit after immersion in water for a 60-day period. **i** CV curves of the unit at various scan rates. **j** CV curves of the unit at various applied loads. **k** Nyquist spectra of the units with different electrodes for MXene, MH (30wn), MH (30), and MH (30)-IL

The proposed pressure-sensing unit offered ultrahigh sensitivity and water stability when pressure and wave disturbances were applied. Its hierarchical structure is shown in Fig. 4a. The MH(30)-IL hydrogel was formed by squeezing the precursor solution into the Cu foam current collector and subsequently solidifying it on the foam pores to serve as an electrode to clamp a poly(vinyl alcohol)/polyvinylpyrrolidone-vitriol (PVA/PVP-H_2_SO_4_) hydrogel dielectric layer with a surface microstructure. The outermost layer of the sensing unit was sealed with a polydimethylsiloxane (PDMS) waterproof film. Herein, the critical steps involved in designing the encapsulation layer were as follows: (i) the encapsulation layer prevented the invasion of underwater Na^+^, K^+^, and Cl^−^, ensuring ample redox reactions at the interface; (ii) the electrode lead wire was not affected by seawater interference; and (iii) the MH(30)-IL electrodes were isolated from corrosion by seawater to increase the service life of the device. In this sandwich design, the MH(30)-IL hydrogel, which had a high interfacial capacitance originating from its fast faradic reactions with acidic electrolytes^35,36^, served as an electrode to guarantee ultrahigh underwater sensitivity and greater water stability in comparison with pure MXene. This difference resulted from the following features: (i) insertion of the HrGO nanosheets suppressed self-restacking of the MXenes, thereby expanding the ion-accessible surface area and exposing adequate reactive functional groups; (ii) the surface perforation design set aside sufficient ion transport channels, resulting in a shorter ion migration path than those seen for the interlayers without internal holes; and (iii) after undergoing cross-linking reactions and IL modification, the oxidation resistance of the MXene was improved substantially, which maintained the activity and enabled sensitive pressure detection even in aquatic environments. Another feature required to ensure stable operation of the device is that the hydrogel dielectric had to exhibit suitable mechanical properties; that is, it should be strong enough to withstand underwater pressures and resilient enough to adapt to frequent shuttling of carriers at different water depths. For these purposes, we fabricated a double-network hydrogel that consisted of a PVA/PVP polymer matrix and H_2_SO_4_ electrolyte (H_2_SO_4_ is an electrolyte that stimulates the MXenes to produce high interfacial capacitance). Specifically, compared with those of single-network PVA hydrogels, the presence of PVP, a complementary component with larger side chain groups, was cross-linked with PVA chains via hydrogen bonding. In addition, ketal formation led to electrophilic rearrangement between the carbonyl groups of the PVP chains and the hydroxyl groups of the PVA chains, which resulted in covalent bonds and the formation of a highly entangled network that enhanced the toughness and mechanical strength of the hydrogel (Fig. 4b)^37,38^. We measured the stress‒strain capacities of the hydrogels with and without PVP and found that the addition of PVP led to great increases in the stiffness, elongation, and fracture strength of the hydrogel, as shown in Fig. S12 (Supplementary Information). Moreover, the resilience of the double-network hydrogel was verified by its ability to withstand a 50% compressive strain after 100 continuous cycles of compression-relaxation (Fig. S13, Supplementary Information), and its ultimate stress had decreased by only 10.2% at the end of the test. The outstanding high resilience of these materials arose from reversible interactions (hydrogen bonding) between the polymer matrices; that is, during the stretching process, breaking and reformation of reversible bonds played pivotal roles in generating exceptional mechanical resilience^39^.

Figure 4c shows that the sensing mechanism of the proposed device included an initial stage and a loading process. The electrical response stemmed from the pseudocapacitive effect, which was primarily generated by the reversible redox reaction occurring at the interface between the electrodes and the dielectric layer. As shown in Fig. 4c(i), *C*_*pse*_ and *R*_*s*_ are the equivalent pseudocapacitance and internal resistance, respectively. When the electrode gradually came into contact with and persisted in compressing the intermediate hydrogel, the H^+^ ions in the dielectric layer migrated between the MXene layers, where they underwent continuous reversible redox reactions with the titanium atoms (Fig. 4c(ii), *x*, *y* = 0, 1, 2, and *β* is the number of electrons in the reaction). With regard to the high specific capacitance of the MH(30)-IL electrodes (for which the relevant tests are described in detail below), the sensitivity (*S*) significantly increased with notable changes in the interfacial capacitance (Δ*C*) because of $S=\delta (\varDelta C/{C}_{0})/\delta P$, where *C*_0_ and *P* are the initial capacitance and applied pressure, respectively. It is worth noting that reversible redox reactions occurred within the nanoscale-thick contact interface; hence, the changes in the interfacial capacitance depended on the contact area (Δ*A*), as described below:

1 $$\Delta C=UAC\times \varDelta A$$

where UAC is the unit area capacitance, which is primarily determined by the inherent properties of the material, such as the density, specific surface area, and ion type, and can be considered a constant^40^. We designed hemispherical microstructures on the surface of a PVA/PVP-H_2_SO_4_ hydrogel by using a preset template for casting and peeling (Fig. S14, Supplementary Information). This was done because the classical mechanical model of hemispherical elastic deformation has been well established^41^; apart from that, the geometric parameters of the hemispherical microstructure were adjusted to meet the required resolution and linearity of the device. The prepared hydrogel dielectric layer with a hemispherical microstructure (with a diameter of 300 μm) is shown in Fig. S15 (Supplementary Information). Accordingly, the applied pressure of the hemispherical microstructure, determined by Δ*A*, can be expressed as follows^42^:

2 $$P=\frac{4E}{3(1-{\nu }^{2})\rho }{\left(\frac{\Delta {A}^{3}}{n{\pi }^{3}}\right)}^{\frac{1}{2}}$$

where *v* and *E* are Poisson’s ratio and Young’s modulus, respectively, for the hydrogel dielectric layer. *ρ* and *n* are the radius of curvature of a hemisphere and the number of hemispheres on the surface of the hydrogel, respectively. The detailed derivation is described in Note S1 (Supplementary Information), and the distribution deformation of the hydrogel with a hemispherical microstructure was determined by finite element analysis (FEA) in Fig. S16 and Movie S1 (Supplementary Information).

The high sensitivity of the sensing unit within a wide pressure range (especially in the mid-pressure region where most fish reside) was the determining factor for detecting weak underwater pressures. The capacitance-pressure curve was clearly composed of three parts (Fig. 4d). In the initial contact stage (<200 kPa) of the hemispherical microstructure and the electrode, the sensitivity reached 7988 kPa^−1^. After that, the sensitivity surged to 136,207 kPa^−1^ within 200–800 kPa, indicating that sufficient contact occurred between the intermediate hydrogel and the MH(30)-IL electrodes to produce interfacial faradic pseudocapacitance. With further pressure applied, the amount of unreactive MH(30)-IL remaining inside the Cu foam gradually decreased, and the sensitivity decreased to 47,423 kPa^−1^; however, this value still remained relatively high compared to those of existing capacitive sensors (Table S2, Supplementary Information). To verify the influence of HrGO intercalation on the fabricated device, various maximum sensitivities were measured by changing the weight fraction (for pure MXene, MH (30wn), 10, 30, or 50 wt %), as exhibited in Fig. S17 (Supplementary Information). By embedding the HrGO nanosheets between neighboring Ti_3_C_2_T_x_, the presence of more ion-accessible active sites induced stronger redox reactions, which led to much greater sensitivities than those of pure MXene (26,819 kPa^−1^). However, the sensitivity of the device initially increased and then decreased as the HrGO concentration was increased (from 38,358 kPa^−1^ at 10 wt % to 41,909 kPa^−1^ at 30 wt % and to 37,379 kPa^−1^ at 50 wt %). This occurred because moderate amounts of intercalants suppressed the self-stacking of the MXenes. In contrast, an excessive HrGO concentration substituted for the MXene as the main reactant in the electrodes, leading to interfacial reactions dominated by the electron double layer (EDL) effect rather than the pseudocapacitance^43^. Another key factor affecting the sensitivity was the concentration of the IL, as it determined both the electrical conductivity and the oxidation resistance of the electrode. Fig. S18 (Supplementary Information) shows the sensitivities determined by adjusting the weight fractions of the ILs. With increasing IL concentration, the maximum sensitivity of the device gradually increased from 95,157 kPa^−1^ at 1 mg of EMIM DCA to 9 mg (147,767 kPa^−1^). It was clear that there was no marked change in maximum sensitivity observed even though the amount of added EMIM DCA was increased from 3 to 9 mg, indicating that an excessive IL defense layer did not improve the sensitivity further once it reached the saturation point. Typically, as the other critical factor for sensitivity enhancement, the UAC primarily depended on the electrical conductivity^44^, whereas there were observable differences in the conductivities of electrodes with different components (Table S3, Supplementary Information). Interestingly, the electrical conductivity decreased gradually with the addition of HrGO, which was attributed to its lower conductivity compared to that of pure MXene. Additionally, the presence of epoxy or hydroxyl groups on the partially reduced HGO may have caused a decrease in the conductivity^45^. The added IL increased the conductivities of the obtained samples due to the high electrical conductivity of the IL itself, as well as the barrier effect that protected the surface of the MXene from forming nonconductive TiO_2_. In general, the reasons for the increased sensitivity of the hybrid hydrogel electrodes included the following: (i) the many active sites on the MXene surface increased the intensity of the interfacial redox reactions; and (ii) after modification with HrGO and the IL, the hybrid hydrogel electrode overcame the defects of pure MXene materials, which easily self-restack and undergo oxidative degradation. It is worth noting that the roughness of the dielectric film is crucial for determining the linearity and sensitivity. We compared the response curves of hemispherical microstructures with different diameters (~200 and 300 μm) and random microstructures printed from sandpaper (Fig. S19, Supplementary Information). It was evident that the dielectric layer with smaller hemisphere diameters (~200 μm) had a greater sensitivity in the low-pressure range than the hydrogel with 300 μm diameter hemispheres, although its sensitivity decreased significantly in the high-pressure range. Although the film with a random microstructure had a lower sensitivity than that with a regular microstructure, its overall linear range was wider. This may be due to the distribution of undercuts and grooves on the filling surface, which provided space for uniform deformation of the surrounding structures.

In view of the need to perceive underwater pressure variations, our sensing unit also required high response and relaxation speeds. To test this, we successively applied and removed a 10 kPa weight on the sensing unit, as shown in Fig. 4e, and the ascending and descending times of the unit were extracted as 13 and 15 ms, respectively. A fast response speed is required for real-time tracking of fish locomotion. In most cases, slow-swimming fish tend to cause only faint pressure changes around them; this requires the development of devices with high-pressure resolution, particularly in deep water, as the viscosity of the water inevitably affects the propagation of pressure waves^46^. We conducted a series of tests to verify the ability of the proposed device to recognize small loads. Figure 4f shows a plot of the pressure resolution test at base pressures of 0 kPa (sea level) and 100 kPa (10 m in depth). First, we sequentially placed ultralight objects with weights of 100 mg (10 Pa) on the surface at a 0 kPa base pressure, followed by explicit step-like capacitance increments. When we placed a 1 kg (100 kPa) metal weight on the device with an acrylic gasket (1 cm × 1 cm) to simulate the underwater static pressure, the device exhibited clear capacitance responses to the weak airflow generated by a rubber bulb, which demonstrated the high sensing resolution of the device at various water depths. Long-term high stability is another indispensable prerequisite for an application-oriented flexible device. Figure 4g shows the capacitance test results for our device after undergoing a 10,000 compression-release test at a peak pressure of 1 kPa. Specifically, the capacitances of the device with the MH(30)-IL electrodes remained nearly unchanged, showing very little drift compared to the initial state. In comparison, the devices equipped with pure MXene and MH(30) electrodes exhibited drifts of 2.66% and 1.43%, respectively, in capacitance losses after 10,000 repeated tests (Fig. S20, Supplementary Information). Moreover, we found that the change in the capacitance of MH(30)-IL (596 nF) was significantly greater than those of MXene (74 nF) and MH(30) (210 nF) under the same cyclic peak pressure. As illustrated, a stable and sustainable interfacial pseudocapacitance was observed even during repeated tests, which was likely due to the IL defense layer and the ability of NAC to reduce the formation of TiO_2_. To verify this point, the Ti 2p XPS spectra of the MXene, MH(30), and MH(30)-IL samples were obtained after 10,000 repeated cycles (Fig. S21, Supplementary Information). The content of TiO_2_ in MH(30)-IL (3.6%) was significantly lower than those in pure MXene (29.48%) and MH(30) (6.99%). Figure 4h shows the capacitance‒pressure curves determined for the sensing unit after it was immersed in water for 60 days. Notably, these curves remained nearly the same, with slight fluctuations (<5%) even after 60 days of immersion, confirming stable waterproofing of the sealing layer and the oxidation resistance of the MH(30)-IL electrodes.

Electrochemical characterization of the sensing unit was used to analyze its pseudocapacitance. The device was treated as an all-solid-state supercapacitor and tested in a three-electrode system. Figure 4i displays the cyclic voltammetry (CV) curves determined for the unit at different scan rates, which exhibited similar shapes with obvious cathodic and anodic peaks and inappreciable distortion even as the scan rate increased, suggesting its high rate capability and capacitive nature. The integrated area of the CV profile generated at 50 mV s^−1^ increased with increasing applied loads from 0 to 500 kPa. As illustrated in Fig. 4j, compared to the loaded conditions, the area surrounded by the CV profile is inapparent in the absence of loading pressure since the interfacial contact area was restricted. Conversely, increased pressure enlarged the accessible area and thus stimulated more significant redox reactions. Furthermore, the electrochemical impedance spectroscopy (EIS) curves plotted in Fig. 4k illustrated the ion transport behavior of our unit with different electrodes. The diameter of the small semicircle in the high-frequency region represents the charge transfer resistance (*R*_*ct*_), and the Rct values of MH(30) and MH(30wn) were lower than that of pure MXene. Moreover, the addition of EMIM DCA increased the electrical conductivity of the unit, which led to a noticeable reduction in the *R*_*ct*_. Additionally, after the addition of the HrGO nanosheets, the electrode exhibited a lower diffusion resistance, which was reflected by slopes higher than those of the MXene electrode. This was attributed to the improved efficiency of ion transport and the increased number of active sites.

### Underwater demonstrations of the pressure-sensing unit

**Fig. 5:**
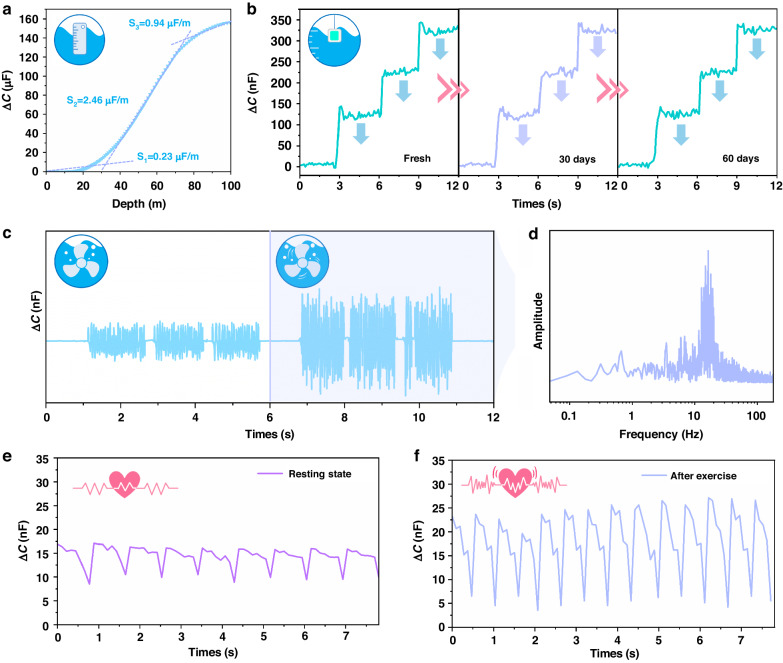
Underwater performance of the pressure-sensing unit. **a** Capacitance-to-depth response. **b** 60-day underwater resolution tests. **c** Speed-adjustable propeller vibration waveforms detected by our device. **d** Frequency domain waveforms of the propeller with high-frequency rotation. **e**, **f** Measurements of the pulse waveforms under resting and exercised states, respectively

We placed the sealed sensing unit in a simulated environment and conducted a series of experiments to verify its underwater detection ability, which is critical for integrating the unit into a highly sensitive underwater vest. Specifically, the sensing unit was placed in a hydraulic test system to simulate aquatic environments at variable depths (Fig. S22, Supplementary Information). Figure 5a shows a plot of the capacitance versus depth, which demonstrated increasing capacitance with increasing water pressure; moreover, the plot was divided into three regions, which was basically consistent with the measurements taken under atmospheric conditions. Additional 60-day resolution tests were also conducted to evaluate the long-term sensing ability of the sensor. The sensor remained submerged in water for an extended period, and it was periodically removed every 30 days before being slowly reimmersed back into the water. As shown in Fig. 5b, the sensor discerned pressure increases of 10 Pa, which is roughly equivalent to a water depth of 1 mm. Although buoyancy affected the increased capacitance caused by the water pressure and resulted in a slightly lower pressure resolution than that seen under atmospheric conditions, the experimental results were sufficient to demonstrate the excellent sensing capability of the device. With high-fidelity pressure resolution, the sensing unit can identify mechanical vibrations underwater. Accordingly, a speed-adjustable propeller with a 5 cm diameter was placed underwater to generate regular water wave disturbances. The water waves propagated toward the side walls of the tank attached to our unit, and the distance traveled was ~10 cm. We first adjusted the propeller to provide low-frequency rotations and activated it three times in succession. Despite the attenuation of the vibrational waves propagating underwater, the capacitance signal was clearly divided into several repeating waveforms. Subsequently, the propeller was adjusted to the high-frequency rotational mode, which resulted in a significant increase in the amplitude of the capacitance output (Fig. 5c). By conducting a time-frequency transformation on the capacitance waveform detected in the high-frequency region, we clearly distinguished the rotational frequency of the propeller at ~16 Hz (Fig. 5d), which demonstrated the fast response speed and superior resolution of our device. More importantly, the sensing unit showed the ability to monitor physiological data under aquatic conditions due to its high resolution. We attached the device to the wrist of a healthy adult volunteer via a biocompatible adhesive and conducted underwater monitoring of the pulse waveforms of the radial artery. (Fig. S23 and Movie S2, Supplementary Information). Figure 5e, f displays the results of the sensing unit for the resting and exercised states of the pulse waveform, respectively. After exercise, increases in both the frequency and amplitude of the pulse waveform were observed. Furthermore, even when submerged underwater, the device was able to clearly resolve the detailed features of the pulse, specifically the systolic, reflected systolic, and diastolic peaks (Fig. S24, Supplementary Information)^47^, so it met the standards of commercial pulse sensors. This experiment demonstrated the possibility of using the sensing unit to detect physiological signals from divers, which provides the basis for real-time feedback on clinical parameters such as diver pulse and heart rate.

### Structure and applications of underwater vests

**Fig. 6:**
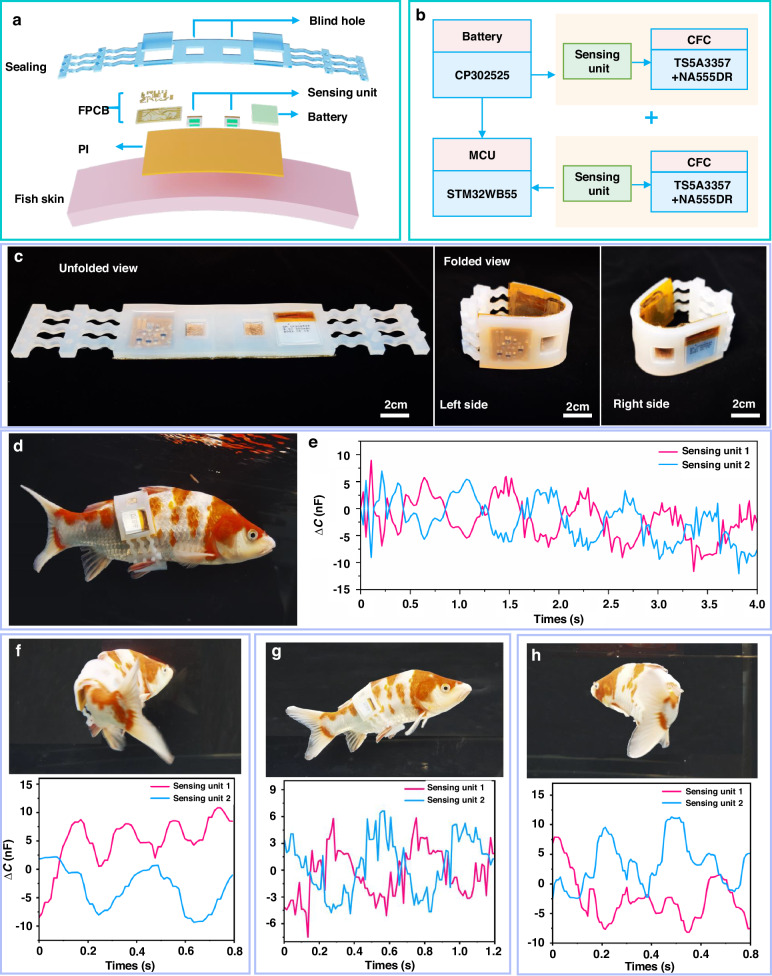
**Structures and applications of the underwater vest.** **a** Exploded-view schematic illustrations of the underwater vest. **b** A block diagram of the underwater vest. **c** Optical images of the unfolded and folded underwater vest. **d** Schematic illustration of an underwater vest attached to a koi fish. **e** Relative capacitance variations captured by the pair of symmetric sensing units. **f** Photographs and corresponding capacitance output for a fish swimming with different locomotion, such as **f** turning right, **g** surfacing, and **h** turning left

The proposed underwater vest offers the remarkable capability to detect disturbance flow fields with high sensitivity and resolution while storing data related to pressure fluctuations over a certain period of time, which provides a long-term, wearable device for determining the behavioral characteristics of fish. In particular, the main structure of the underwater vest comprised (i) a PDMS encapsulation device with a “double-breasted” design and a length that can be adjusted to accommodate different fish sizes; (ii) a pair of sensing units for perceiving the pressure changes caused by oscillations in the symmetric lateral flow fields of fish; and (iii) a dedicated flexible printed circuit board (FPCB) for detecting and storing dual-channel capacitive signals, as shown in Fig. 6a. Accordingly, the entire underwater vest was a conformal device that could be attached to fish via three stacking layers, including a waterproof and stretchable PDMS packaging material as the top sealing layer; an interconnecting series of essential components, which comprised the sensing units, an FPCB and a battery; an intermediate working layer; and a flexible polyimide (PI) layer as the bottom substrate. The soft package embedded within the working layer could be wrapped around the fish like a wearable vest. Specifically, the serpentine structures on both sides of the encapsulation provided stretchability. In addition, the hollow design accommodated the fish fins, ensuring that our device could be attached firmly to the fish. Notably, blind holes with embedded sensing units were designed inside the encapsulation system based on the following considerations: (i) the blind holes prevented surrounding shear forces from interfering with the sensing units, thus allowing only purely normal pressures to be detected; and (ii) the sensing units enclosed within the encapsulation layer may undergo deformations when the packaging is bent or stretched, resulting in extraneous pressure interference. To verify the mechanism by which the blind holes isolated the impact of encapsulation deformation and protected the sensing units from extraneous interference within them, we used FEA and investigated the deformation mechanisms of the structures with and without blind holes when subjected to the same bending conditions (Fig. S25, Supplementary Information). In the absence of surface blind holes during encapsulation, the stress generated by deformation was relatively evenly distributed across different regions. In sharp contrast, the stress inside the blind holes was significantly reduced, creating a stable area suitable for the sensing units (Fig. S26, Supplementary Information). Furthermore, the above simulation results were supported by bending tests. Specifically, the sensing units were gradually bent to 90°, and their capacitance changes were recorded (Fig. S27a–c, Supplementary Information). Without blind hole protection, the unit capacitance increased by ~1000 nF. In contrast, the capacitance changes in the two units embedded in blind holes remained within the range of 1 nF, indicating the shielding effect of the blind holes on bending stress (Fig. S27d, Supplementary Information). Another practical concern arose from the influence of coupled factors such as eddies and ocean currents, which may interfere with the flow fields around fish generated by locomotion. Based on these considerations, we analyzed the surrounding flow fields and designed the locations of the sensing units accordingly. Notably, we symmetrically deployed a pair of sensing units on both sides near the back of the fish. This was chosen because the swing amplitudes at the position near the fish back are relatively small. The deployment of these devices avoided potential deformations, such as bending effects, on the units and enabled them to detect changes in the water pressure caused by fish locomotion. Subsequently, we developed a numerical simulation of fish swimming with fluid mechanics. In performing FEA, the water was modeled as a fluid, and the fish were modeled as solids with a certain degree of displacement^48^. The sizes and relative position parameters of the fish and the sensing units are shown in Figure S28a (Supplementary Information). Fluid–structure interaction (FSI) coupling was established to analyze the interaction mechanism between these two entities (Figure S28b and Note S2, Supplementary Information). To address the problems arising from changes in the fish shape and position and their effects on the surrounding water, we employed the arbitrary Lagrangian–Eulerian (ALE) method in COMSOL Multiphysics software^49^ (Movie S3, Supplementary Information). As shown in Fig. S29 (Supplementary Information), when the fish swam smoothly in a uniform flow, the pressure on the symmetric sides followed a predictable pattern, with nearly equal magnitude but opposite signs. This result was used to guide the placement of the sensing units; that is, we inferred the motion states of fish by setting symmetric sensing units. Even if interference from eddies or ocean currents operated in a particular direction, the sensing units could not detect regular waveforms similar to those generated by fish swimming movements.

A block diagram of the sensing units accompanied by the data acquisition and processing system is shown in Fig. 6b. The interfacial capacitance of the sensing units reached up to several hundred μF, which was determined by converting that value into a frequency^50^. A 555 precision timer-based capacitance-frequency conversion (CFC) circuit was designed to read the dual-channel data collected by the sensing units (see the details in Fig. S30, Supplementary Information). Subsequently, the microcontroller (MCU) implemented the calculation and storage of the pressure signals. Moreover, we demonstrate the complete strategy for fabricating an underwater vest in the three-dimensional diagram in Fig. S31 (Supplementary Information). The details of the entire fabrication process and the materials used are described in the Methods Subsection. Figure 6c shows optical images of an underwater vest with unfolded and folded views, and the overall size was 21.5 × 3.5 × 0.6 cm when it was unfolded. We also demonstrated the attachment mechanism of our underwater vest by bonding it to a koi fish (Fig. 6d and Fig. S32, Supplementary Information), in which the component affixed to the right side of the fish was labeled sensing unit 1, and the other unit on the left side was labeled sensing unit 2. After that, the koi fish wearing our vest were released back into the aquarium (with a swimming depth range of approximately 10–30 cm). The natural locomotion of the fish was minimally limited due to the flexibility, conformability, and ultralightweight mass of the vest (the entire structure with embedded components weighted <8 g). Notably, we monitored fish activities and behaviors with an underwater camera, which allowed us to analyze and compare the hydrodynamic pressure field data recorded by the proposed system (Movie S4, Supplementary Information). We recorded the swimming of the fish over a period of time with a pair of symmetrical pressure-sensing units, as shown in Fig. S33 (Supplementary Information). Furthermore, four typical locomotion patterns of the fish were identified with the monitoring footage. Specifically, Fig. 6e displays variations in the symmetric lateral pressure waves of a fish, which corresponded to its regular tail-swishing motion accompanied by sinking. As shown in Fig. 6f–h, three typical locomotion motions were identified by the capacitance outputs of the paired sensing units, i.e., turning right, surfacing, and turning left. Specifically, as the fish turned to the right side, the pressure applied to sensing unit 1 noticeably increased, whereas the capacitance of unit 2 decreased. Additionally, synchronized increases in the relative capacitance values of the paired units reflected the rhythmic slow, upward swimming activity of the fish. Similarly, when the fish turned left, the normal pressure on the left side was significantly greater than that applied to the right side. The complete capacitance fluctuation of the fish turning left is shown in Fig. S34 (Supplementary Information), and Fig. 6h displays the instantaneous turning action captured above. As we stimulated the smooth swimming of the fish with a light, it instinctively accelerated and evaded it. The fish body underwent significant swinging, which caused a surge in the surrounding flow field. Once the fish had escaped the disturbance or returned to a calm state, we observed that locomotion became slower and that the swinging cycles decreased. The process was fully recorded by the underwater vest (Fig. S35, Supplementary Information). Hence, the underwater vest with superior sensitivity and resolution provides a path for continuously perceiving variations in hydrodynamic pressure around a fish and thus inferring matching locomotion and behaviors via a wearable tagging method. Notably, this approach can be used for posture recognition with koi and extended to other carrier platforms. Owing to the wide working range of the sensing units, the proposed device showed good potential for expanded use in marine environments. In other words, underwater vests may aid in monitoring the locomotion of nearshore aquatic fish, such as yellow croaker and grouper, suggesting that underwater vests could be useful for ethology research and marine ecosystem observation. In the future, advanced aquatic devices could even be used in marine soft robots to allow feedback from the surrounding flow fields to control motion.

## Conclusion

In summary, we developed a wearable and water-stable locomotion awareness device, referred to as an underwater vest, to recognize the surrounding flow fields caused by fish swimming and their specific locomotion and thus inferred the adaptive behavioral characteristics of the fish; this device is a promising ethology research tool for tracking in the natural aquatic environment. Importantly, the core components of the conformal and noninvasive tagging electronic device included a pair of pseudocapacitive pressure-sensing units symmetrically embedded in blind holes, which consisted of MH(30)-IL hydrogel electrodes and a PVA/PVP-H_2_SO_4_ double-network hydrogel dielectric layer. Notably, the active hydrogel electrodes were prepared by cross-linking HrGO and MXene with the aid of NAC and further modifying them with EMIM DCA. The interactions of HrGO between the MXene lamellae effectively suppressed self-stacking, and highly conductive ILs constructed a unique protection mechanism to prevent degradation of the MXene with oxidizing electrolytes or in a long-term humid environment. Due to its water stability and high interfacial capacitance, the pressure-sensing unit exhibited an ultrahigh sensitivity up to 136,207 kPa^−1^, high underwater resolution, and long-term underwater stability. The superior aquatic sensing performance of our units enables the use of an integrated underwater vest to achieve sensitive detection of minor vibrational waves at a range of water depths. The potential applications of underwater vests are promising and attractive; additionally, the underwater vests can be used for recording fish movements and migration routes and even for monitoring weak flow fields, such as seismic waves and ocean currents.

## Materials and methods

### Preparation of the Ti_3_C_2_T_x_ and HGO dispersion

Briefly, 1 g of lithium fluoride (LiF) powder (99%, Aladin) and 1 g of Ti_3_AlC_2_ powder (Xiyan Technology Co., Ltd.) were immersed in 20 mL of 9 M hydrochloric acid (Sinopharm Chemical Reagent Co., Ltd., China) solution and stirred at 35 °C for 24 h. The obtained products were rinsed several times with deionized (DI) water and then centrifuged at 3500 rpm for 5 min until the pH was close to neutral. Subsequently, 25 mg of the precipitate was weighed and dispersed in 5 ml of DI water, followed by sonication for 3 h under an argon-protective atmosphere to form a colloidal suspension of MXene at a concentration of 5 mg mL^−1^. Specifically, 1 mL of 30 wt% H_2_O_2_ aqueous solution (Sinopharm Chemical Reagent Co., Ltd.) was dispersed into a GO dispersion (50 mL, 0.4 mg mL^−1^, Tanfeng Technology Co., Ltd.), where H_2_O_2_ etched the holes on the GO nanosheets. The mixture was subsequently transferred to an autoclave and kept at 100 °C for 5 h. The above solution was repeatedly filtered and washed with DI water to remove the residual H_2_O_2_. The washed precipitate was then freeze-dried to obtain HGO films, and 0.01 g of the precipitate was dispersed into 5 mL of DI water with ultrasonic irradiation to obtain a 2 mg mL^−1^ HGO solution.

### Synthesis of the MH-IL hydrogel and MH-IL hydrogel-derived film

Typically, an HGO solution (4.5 ml, 2 mg ml^−1^), an MXene colloidal suspension (4.2 ml, 5 mg ml^−1^), and NAC (3 mg, 99%, Aladdin) were mixed by magnetic stirring and ultrasonic irradiation for 30 min under Ar to obtain a homogeneous suspension. Subsequently, the EMIM DCA ionic liquid (1 mg, 98%, Aladin) was added to the above homogeneous suspension and stirred at room temperature for 1 h, followed by heating at 150 °C for 30 min under an Ar atmosphere to reduce the GO thermally. After that, the suspension was sealed in a vial and kept at 70 °C for 4 h to complete the polymerization reaction and obtain the MXene/HrGO-IL (MH-IL) hydrogel (the detailed contents of the proposed hydrogel are illustrated in Supplementary Table 1). Notably, the MH-IL precursor solution obtained prior to polymerization was vacuum-filtered through a nylon membrane. After drying at room temperature, the mixture was peeled off to obtain an MH-IL hydrogel-derived film.

### Preparation of a double-network hydrogel dielectric layer

Briefly, 1.7 g of poly(vinyl alcohol) (PVA, 99 mol%, Aladin) and 0.7 g of polyvinylpyrrolidone (PVP, Mw~55,000, Aladin) were added to 8 ml of 1 M vitriol (H_2_SO_4_, Sinopharm Chemical Reagent Co. Ltd.) via strong magnetic stirring at 85 °C for 2 h and sonicating for 5 min until the PVA and PVP were completely dissolved. The obtained mixture was then poured into a custom-made epoxy resin preset template with hemispherical depression microstructures (Supplementary Fig. 15), followed by freezing at −50 °C for 20 h. The template was removed and left at room temperature for 2 h. Finally, freeze‒thawing was performed four times to fabricate the tough double-network PVA/PVP-H_2_SO_4_ hydrogel.

### Construction of the pressure-sensing unit

To mount the electrodes firmly on the collectors, the MH-IL precursor solution was applied evenly to a 15 mm × 10 mm × 0.5 mm Cu foam with a spatula prior to polymerization. A Cu foam of the same size was used to sandwich the applied active material in the middle, which was squeezed under 8–10 MPa pressures to fully immerse the hydrogel precursor in the pores of the foams. After natural air drying of the two pieces of hydrogel-soaked foams overnight, current collectors with MH-IL hydrogel electrodes were prepared. The double-network hydrogel dielectric layer was sandwiched between the upper and lower Cu foams. Subsequently, PDMS precursors (Sygard 184, Dow Corning) were applied to the Cu foams, which were spin-coated at 600 r min^−1^ for 1 min. After solidifying at 90 °C for 5 h, sealing layers were fabricated for the sensing unit, and the final highly sensitive sensing unit was obtained.

### Finite element analysis

FEA of the deformation distributions of hydrogels with hemispherical microstructures was conducted by utilizing the commercial software Abaqus 2019. The Cu foam with the MH-IL electrode was treated as rigid with Young’s modulus of *E* ~3.8 GPa. The PVA/PVP-H_2_SO_4_ hydrogel was modeled as a neo-Hookean superelastic material with a Poisson’s ratio of 0.5 and an *E* of ~8 MPa. FEA of the encapsulation deformations with and without blind holes were also performed with Abaqus 2019, in which the material properties were set to Poisson’s ratio = 0.5 and *E* ~ 2.25 MPa.

The FEA of the surrounding flow field triggered by fish swimming was conducted with COMSOL 6.1 Multiphysics software. Changes in the shape and position of the fish caused deformation of the surrounding water domain; to this end, the arbitrary Lagrangian–Eulerian technique was used to delineate the boundary layer mesh of the FSI. The fish was modeled as a shuttle-shaped uniform material with Poisson’s ratio = 0.33 and *E* ~ 0.2 MPa.

### Construction of the underwater vest for the data acquisition and processing system

A flexible printed circuit board was prepared on the conductive Cu layer (10 μm thick) of the PI substrate. Low-temperature silver paste (Guangzhou Solderwell Technology Co., Ltd.) was used to connect the sensing units and the data acquisition and processing system, which included a 1-channel analog multiplexer (TS5A3357, Texas Instruments), precision timer (NA555, Texas Instruments), MCU (STM32 WB55) and battery (CP302525, Shenzhen Enbar Technology Co., Ltd.).

### Assembly of the underwater vest

To prepare for waterproof encapsulation, PDMS (Sygard 184, Dow Corning, crosslink: PDMS = 1:10) was poured into the preset template, then it was peeled off after curing at 90 °C for 5 h (images of the template and encapsulation are shown in Fig. S36, Supplementary Information). After the integration of all components on the PI substrate, an O_2_ plasma was applied to the encapsulated PDMS, which was subsequently bonded to the PI substrate by a double-sided adhesive. The O_2_ treatment during encapsulation improved adhesion to the substrate and thus prevented water immersion. We compared the waterproofing effects of the encapsulants treated with and without O_2_ plasma. Specifically, a piece of pH test paper was stuffed in the blind hole of the encapsulation plate, which was adhered to the bottom substrate after treatment with the plasma. The encapsulation agent was separated from the substrate after the sample was immersed in a weakly acidic solution for 20 days. The pH test paper did not change color, while the pH test paper inside the sample without the plasma treatment changed significantly, indicating immersion in the acidic water (Fig. S37, Supplementary Information).

### Characterization and measurements

The hydrogel samples were characterized with SEM (Tescan, One Max 50) and TEM (Thermo Fisher Scientific, Talos F200X). The crystalline phases and chemical compositions were determined with XRD (Smartlab SE, *λ* = 0.15406 nm) and Raman spectroscopy (Renishaw spectrometer with a 532 nm laser as the exciting source), respectively. XPS was conducted with a Shimadzu, Axis Supra+ system. The capacitance of the sensing unit was measured with an LCR instrument (IM3536, HIOKI) with a 20 Hz scanning frequency and a 3 V potential. The loading pressure was applied and recorded with an automatic dynamometer (Dongguan Zhiqu Precision Instrument Co., Ltd., 990-zq). A three-axis translation stage (Omtools) was used to test the protein stability. The electrochemical tests were conducted with a PGSTAT302N workstation (Metrohm).

### Experimental settings of the underwater vest

A koi fish with a length of ~30 cm was placed in the sensing vest and used as the experimental subject, and it was placed in a water tank with a depth of ~40 cm. Moreover, an underwater camera (S3, Ezviz Technology Co., Ltd.) was placed in the tank to track changes in the locomotion of the fish.

### Supplementary information

FEA of PVA/PVP-H2SO4 hydrogel with hemispherical microstructures

The pressure sensing unit of measuring the pulse waveform

FEA of the flow velocity around fish

“Underwater vest” of perceiving for fish locomotion

Supplementary Material
