# Supplementary material for: An underwater vest containing an antioxidant MXene hydrogel for sensitive recognition of fish locomotion
Source: Microsyst Nanoeng. 2024 Mar 22;10:41. doi: 10.1038/s41378-024-00675-8 (PMC10957866; doi:10.1038/s41378-024-00675-8)
Supplement: Supplementary file 5 — Supplementary Material [file 41378_2024_675_MOESM5_ESM.docx]

Supporting Information for

**An underwater vest containing an antioxidant MXene hydrogel for sensitive recognition of fish locomotion**

Chengxiu Yang, Jiafei Hu*, Lihui Liu, Shaowei Wu, Mengchun Pan, Yan Liu, Haomiao Wang, Peisen Li, Qi Zhang, Weicheng Qiu, Huihui Luo

*College of Intelligence Science and Technology, National University of Defense Technology, Changsha 410073, China*

**Table S1.** The contents of MXene, HrGO, NAC and EMIM DCA for various samples of this work.

|  | **MXene suspension**  **(ml)** | **HGO solution**  **(ml)** | **NAC**  **(mg)** | **EMIM DCA** |
| --- | --- | --- | --- | --- |
| MXene | 6 | 0 | 0 | 0 |
| MH (10) | 5.4 | 1.5 | 3 | 0 |
| MH (20) | 4.8 | 3 | 3 | 0 |
| MH (30) | 4.2 | 4.5 | 3 | 0 |
| MH (40) | 3.6 | 6 | 3 | 0 |
| MH (50) | 3 | 7.5 | 3 | 0 |
| MH (30wn) | 4.8 | 4.5 | 0 | 0 |
| MH (30)-IL(1) | 4.2 | 4.5 | 3 | 1 |
| MH (30)-IL(3) | 4.2 | 4.5 | 3 | 3 |
| MH (30)-IL(9) | 4.2 | 4.5 | 3 | 9 |

The concentrations of MXene suspension and HGO solution are 5 mg ml^-1^ and 2 mg ml^-1^, respectively.

**Table S2.** Comparison of the performance with representative capacitive mechanism sensors.

| **Type** | **Maximum sensitivity**  **(kPa^-1^)** | **Pressure range**  **(kPa)** | **Ref.** |
| --- | --- | --- | --- |
| Capacitive | 10.2 | 0-100 | 1 |
| Capacitive | 6.583 | 0-1 | 2 |
| Capacitive | 0.39 | 0-25 | 3 |
| Capacitive | 0.047 | 0-200 | 4 |
| Capacitive | 0.51 | 0-400 | 5 |
| Capacitive | 3.13 | 0-50 | 6 |
| Iontronic | 1.3 | 0-15 | 7 |
| Iontronic | 5.5 | 0-250 | 8 |
| Iontronic | 174 | 0-400 | 9 |
| Iontronic | 365 | 0-1000 | 10 |
| Iontronic | 33.16 | 0-176 | 11 |
| Iontronic | 3302.9 | 0-360 | 12 |
| Iontronic | 9280 | 0-114 | 13 |
| Pseudocapacitance | 162.9 | 0-160 | 14 |
| Pseudocapacitance | 3155.4 | 0-10000 | 15 |
| Pseudocapacitance | 46730 | 0-14000 | 16 |
| This work | 136207 | 0-10000 |  |

**Table S3.** Electrical conductivity of the prepared electrodes.

|  | **Electrical conductivity (S cm^-1^)** |  | **Electrical conductivity (S cm^-1^)** |
| --- | --- | --- | --- |
| MXene | 62.1 | MH (40) | 0.32 |
| MH (30wn) | 0.79 | MH (50) | 0.16 |
| MH (10) | 1.23 | MH (30)-IL(1) | 0.92 |
| MH (20) | 0.97 | MH (30)-IL(3) | 1.18 |
| MH (30) | 0.84 | MH (30)-IL(9) | 1.20 |

**Note S1**

The variation in the contact area is determined by the compression deformation of hemispherical array. According to the classical Hertz Contact theory ^17^, the electrode is regarded as a rigid plane, for a single hemispherical bulge, the radius (*r*) of the contact area between it and the electrode during compression can be calculated as:

(1)

where $E_{1}$, $E_{2}$ and $\nu_{1}$, $\nu_{2}$ denote the Young’s modulus and Poisson’s ratios of the elastic bulge and electrode, respectively. $\rho$ and $P_{s}$ are the radius of curvature of a hemisphere and the normal applied pressure, respectively. Due to $E_{2}\gg E_{1}$, Equation 1 can be expressed as:

(2)

and the variation in the contact area of the hemisphere bulge with the electrode ($\Delta A$) can be expressed as:

$\Delta A=n\pi r^{2}=n\pi\left(\frac{3}{4}\rho\left(\frac{1-\nu_{1}^{2}}{E_{1}}\right)P_{s}\right)^{\frac{2}{3}}$ (3)

where $n$ denotes the number of hemispheres on the surface of hydrogel. For the whole hydrogel dielectric layer, the applied pressure *P* can be calculated as:

$P=nP_{s}=\frac{4E_{1}}{3(1-\nu_{1}^{2})\rho}\left(\frac{\Delta A^{3}}{n\pi^{3}}\right)^{\frac{1}{2}}$ (4)

**Note S2**

The interaction between fish and water can be regarded as an issue of coupling combines fluid flow with structural mechanics. The Fluid-Structure Interaction (FSI) coupling can be established through the flow interface model and the structural mechanic interface. Assuming that water can be seen as an incompressible and Newtonian fluid, in which case the Navier–Stokes (N-S) equations are constructed as ^18^:

$\rho_{0}\frac{\partial u_{fluid}}{\partial t}+\rho_{0}(u_{fluid}\cdot\nabla)u_{fluid}=\nabla(pI+\kappa)+F$ (5)

$\rho_{0}\nabla\cdot u_{fluid}=0$ (6)

where $\rho_{0}$ is the density of water, $u_{fluid}$ is the velocity vector of water, $p$ is the pressure, $I$ and $\kappa$ are the identity matrix and viscous stress tensor, respectively, and $F$ is the volume force vector. According to the first Piola-Kirchhoff stress tensor, the equation of fish motion can be written as ^19^:

$\rho_{1}\frac{\partial^{2}u_{solid}}{\partial t^{2}}=\nabla\cdot p^{T}+F$ (7)

$u_{fluid}=u_{solid}$ (8)

where $\rho_{1}$ denotes the density of fish, $t$ is the time the fish swims, and $u_{solid}$ is the velocity vector of fish. The FSI couplings reflect on the boundaries between the water and the fish. Then we used the arbitrary Lagrangian-Eulerian (ALE) method in Comsol Multiphysics software to combine the fluid flow formulated using Eulerian description with solid mechanics formulated using Lagrangian description and material frame.


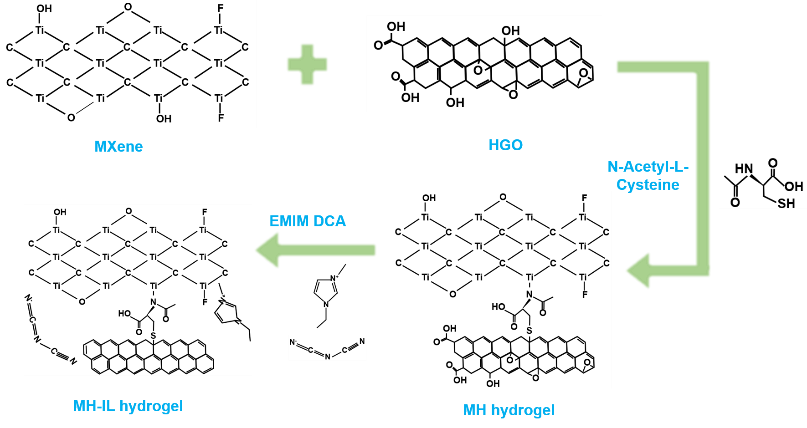


**Figure S1.** Formation mechanism of MH(30)-IL hydrogel.


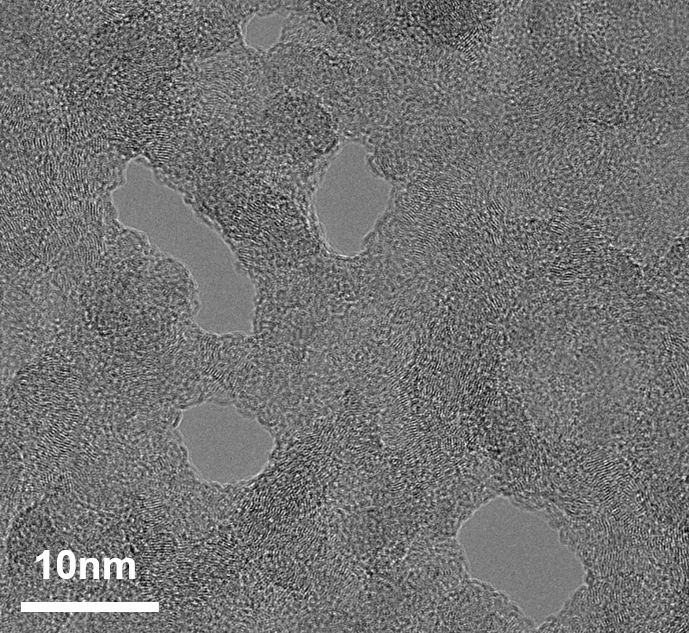


**Figure S2.** Transmission electron microscope (TEM) of holey graphene oxide (HGO).


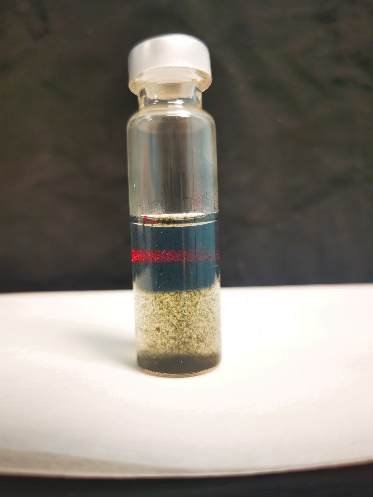


**Figure S3.** Tyndall effect for MXene colloidal suspension.


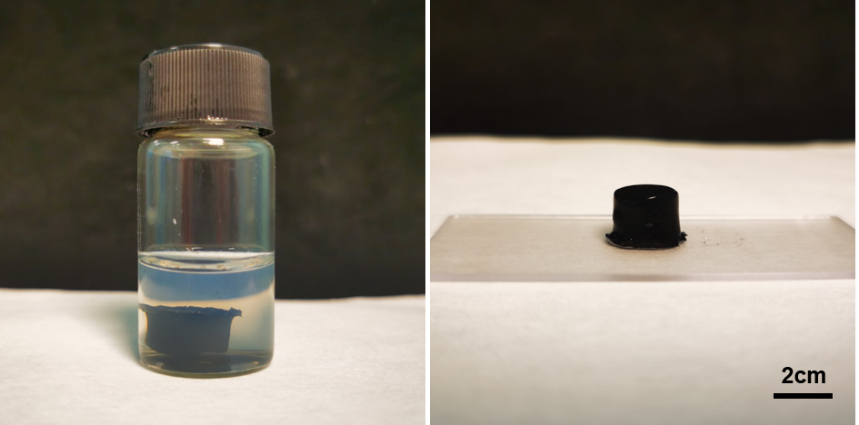


**Figure S4.** The optical images of MH hydrogel.


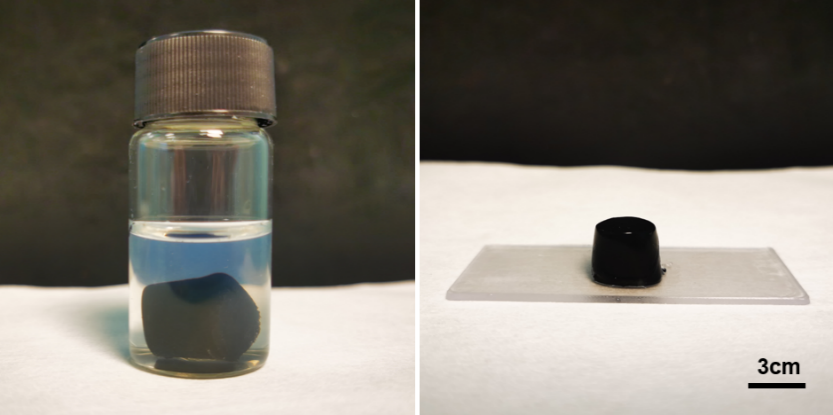


**Figure S5.** The optical images of MH-IL hydrogel.


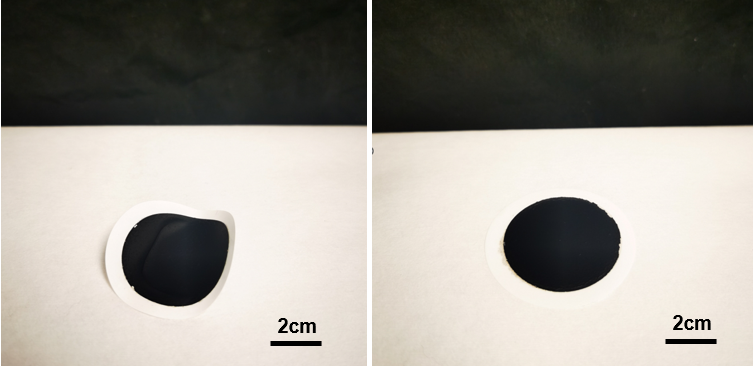


**Figure S6.** The optical images of MH(30), MH(30)-IL hydrogel-derived films.


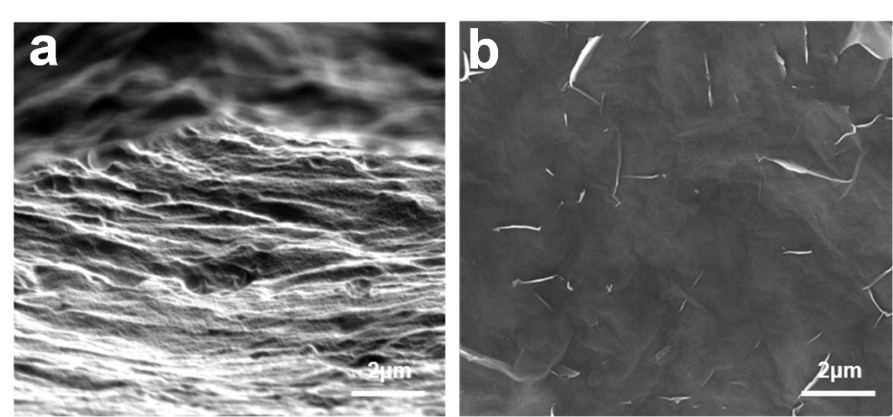


**Figure S7.** Scanning electron microscope (SEM) images of MXene film: (a) cross-sectional, (b) top-view.


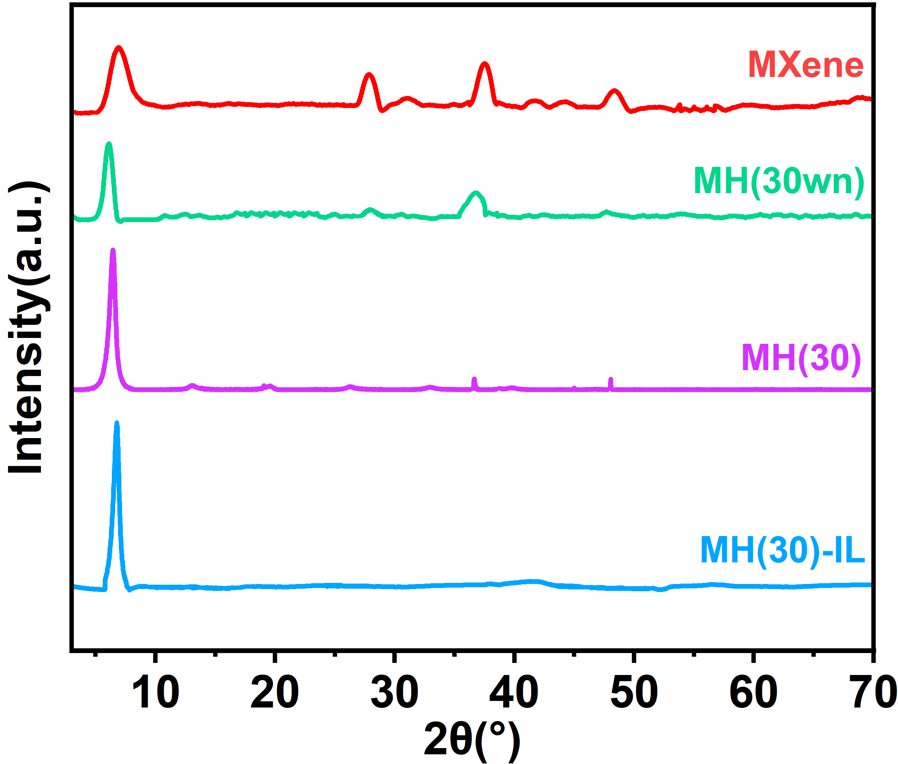


**Figure S8.** XRD patterns of pure MXene, MH (30wn), MH (30) and MH (30)-IL after storing for 90 days.


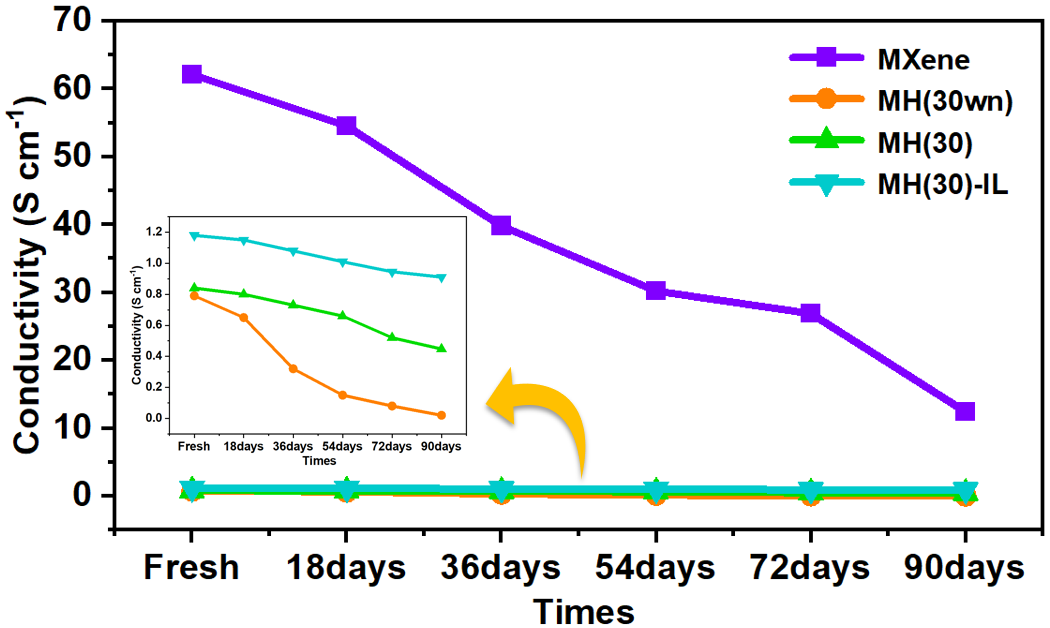


**Figure S9.** Electrical conductivity variations using 4-point probe method of the prepared samples over time.


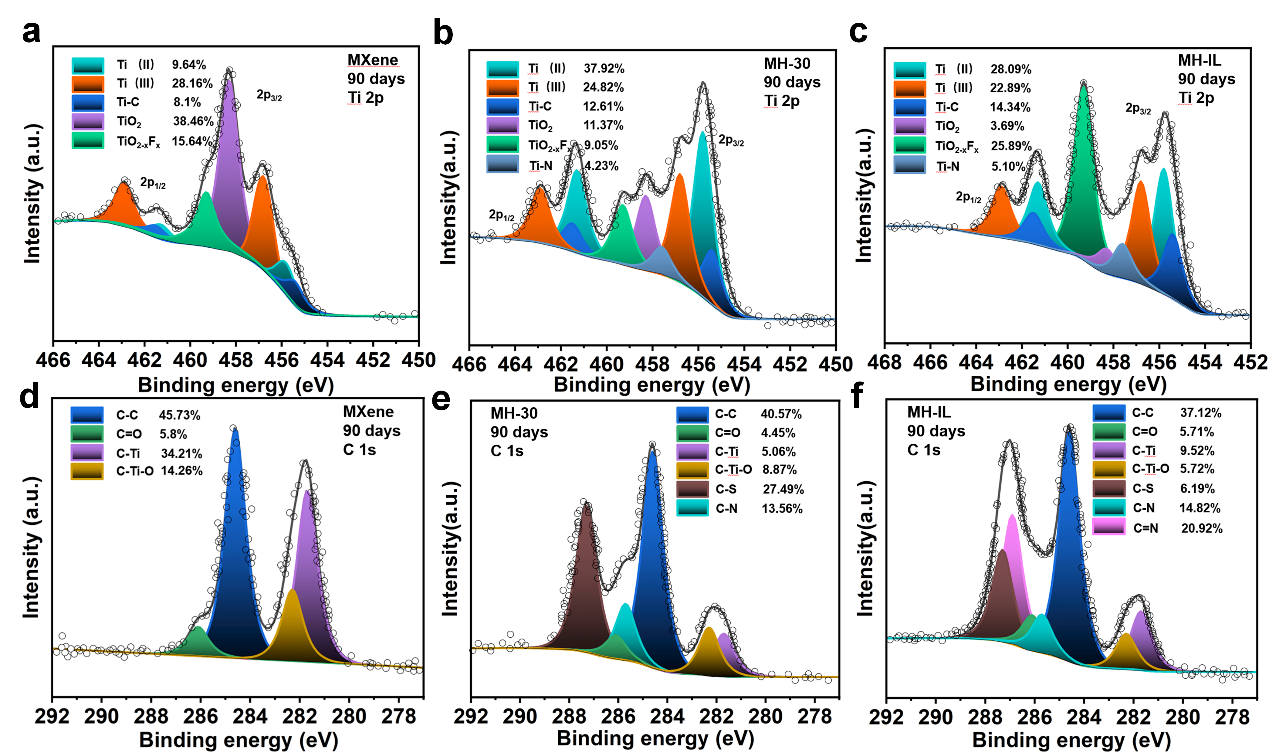


**Figure S10.** X-ray photoelectron spectroscopy (XPS) spectra of Ti 2p for (a) MXene, (b) MH(30), (c) MH(30)-IL being stored in water for 90 days. C 1s for (d) MXene, (e) MH(30), (f) MH(30)-IL being stored in water for 90 days.


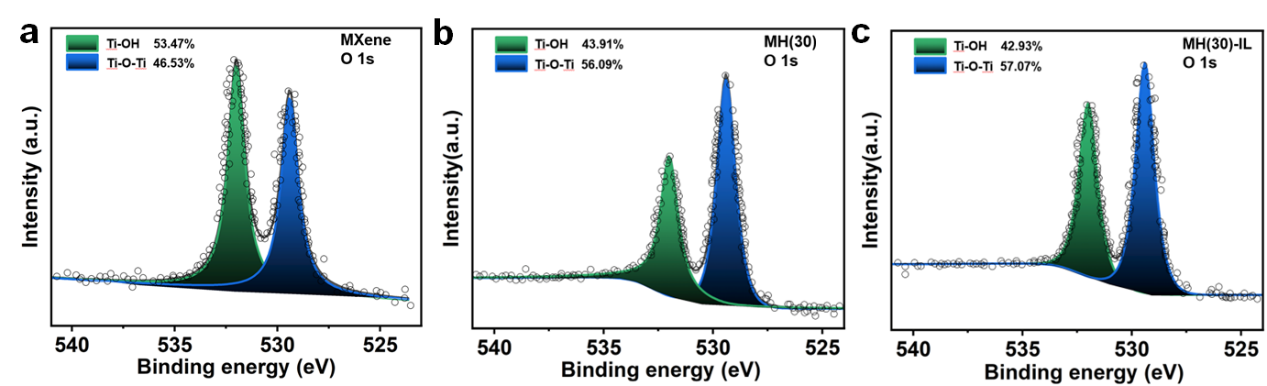


**Figure S11.** XPS spectra of O 1s for (a) MXene, (b) MH(30), (c) MH(30)-IL, respectively.


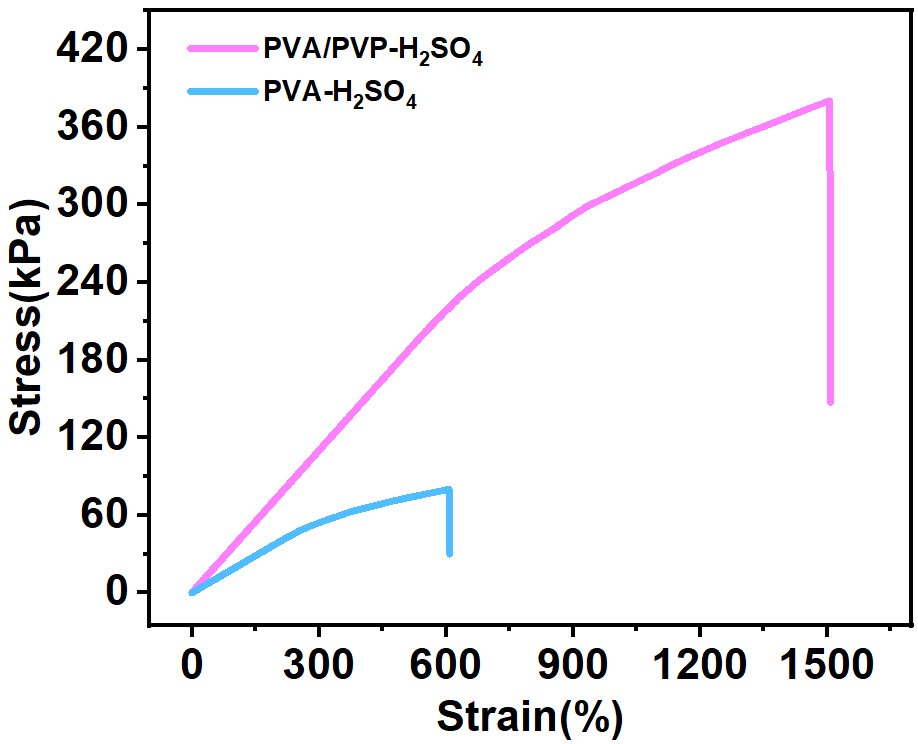


**Figure S12.** Stress-strain curves of the hydrogels with and without PVP.


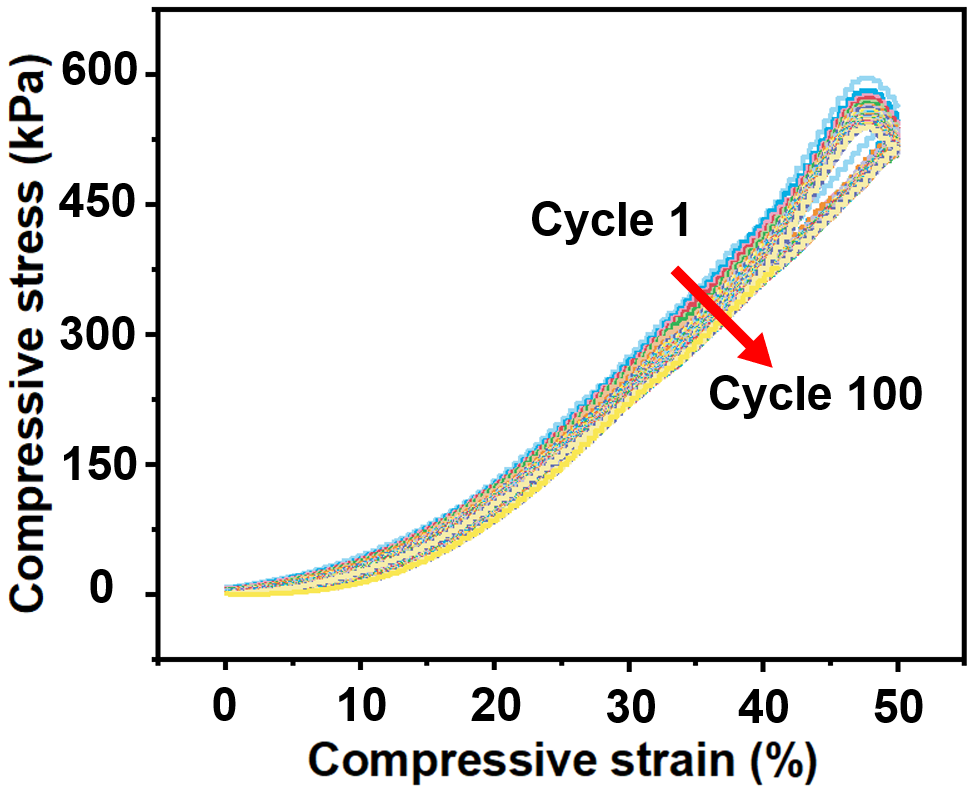


**Figure S13.** Stress-strain curves for hydrogels of 100 cycles of the loading and unloading process.


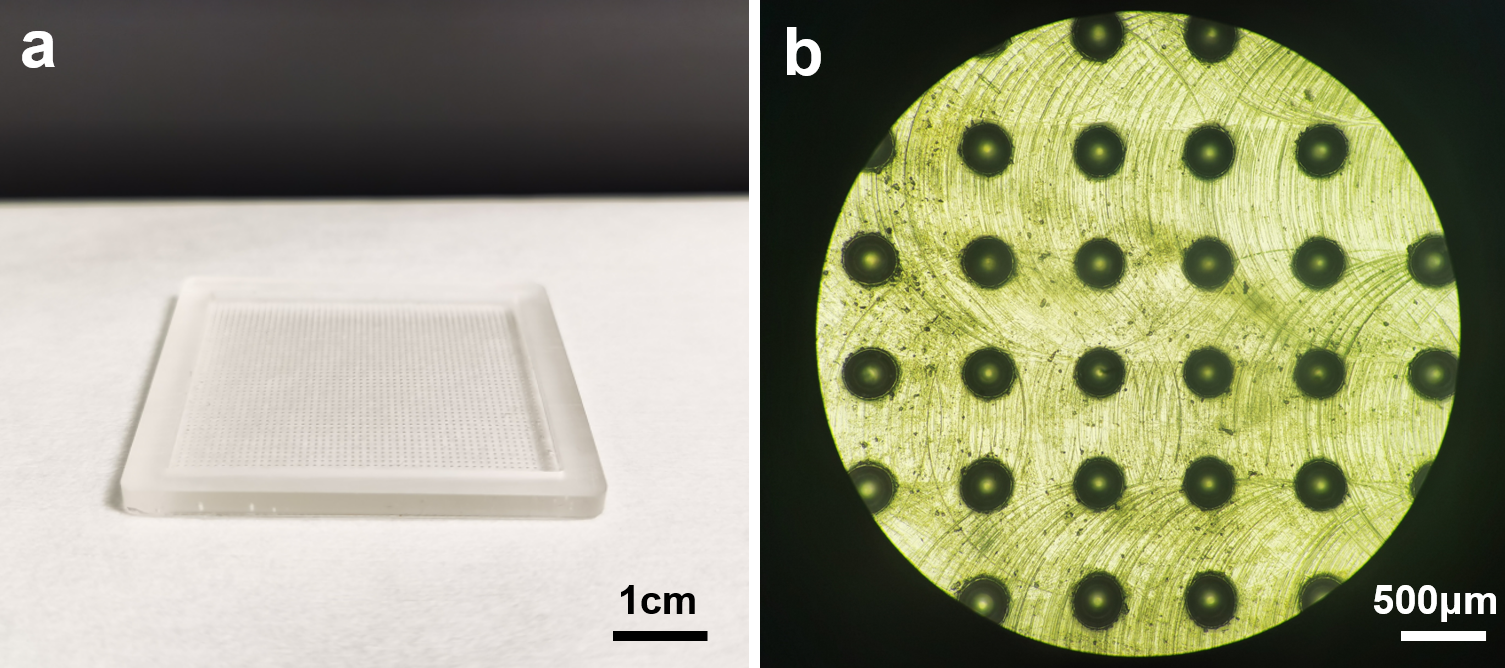


**Figure S14.** The preset template of preparing the PVA/PVP-H_2_SO_4_ hydrogel for (a) optical photograph, (b) optical microscope photograph.


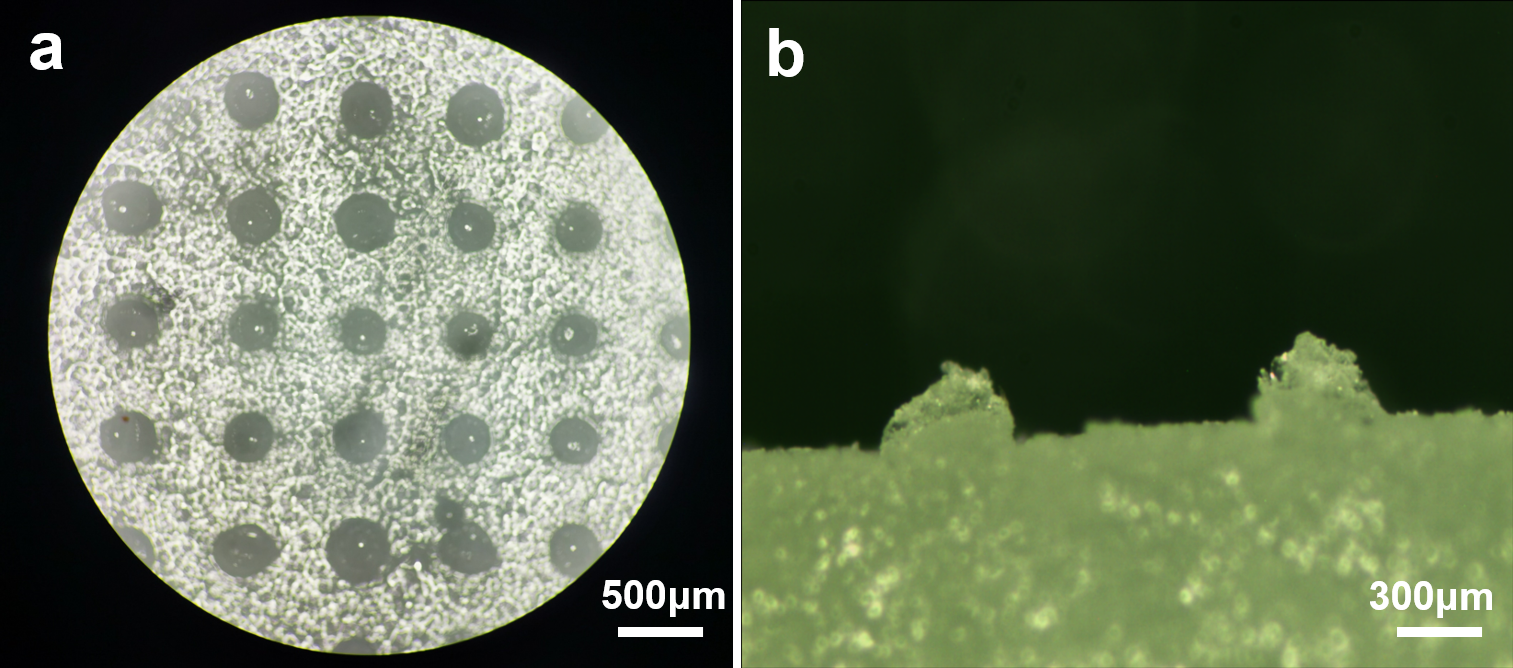


**Figure S15.** The optical microscope photograph of PVA/PVP-H_2_SO_4_ hydrogel for (a) top-view, (b) cross-sectional view.


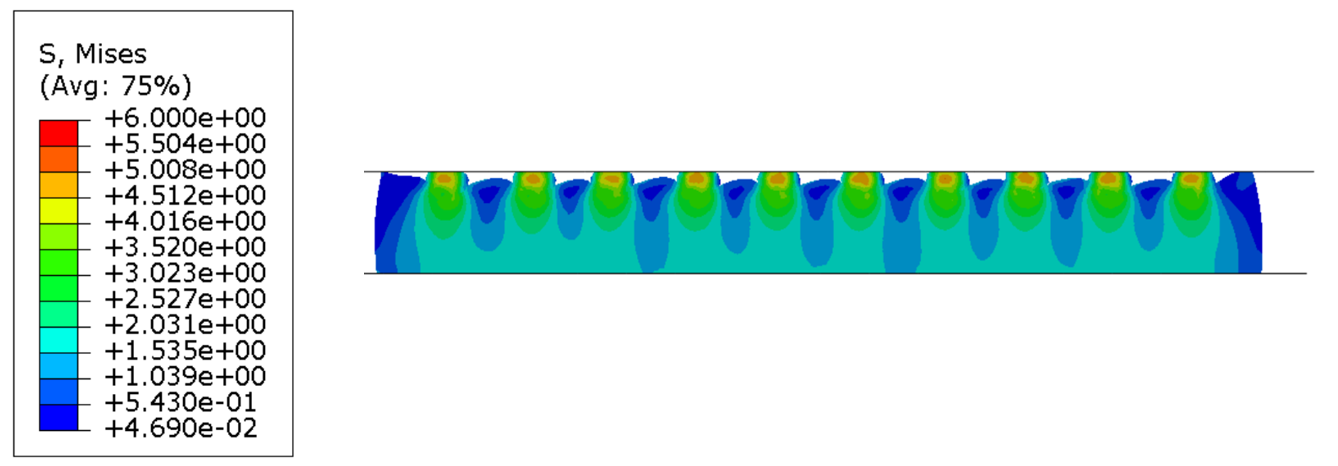


**Figure S16.** The simulated results of the PVA/PVP-H_2_SO_4_ hydrogel with elastic hemispherical microstructures in contact with electrodes.


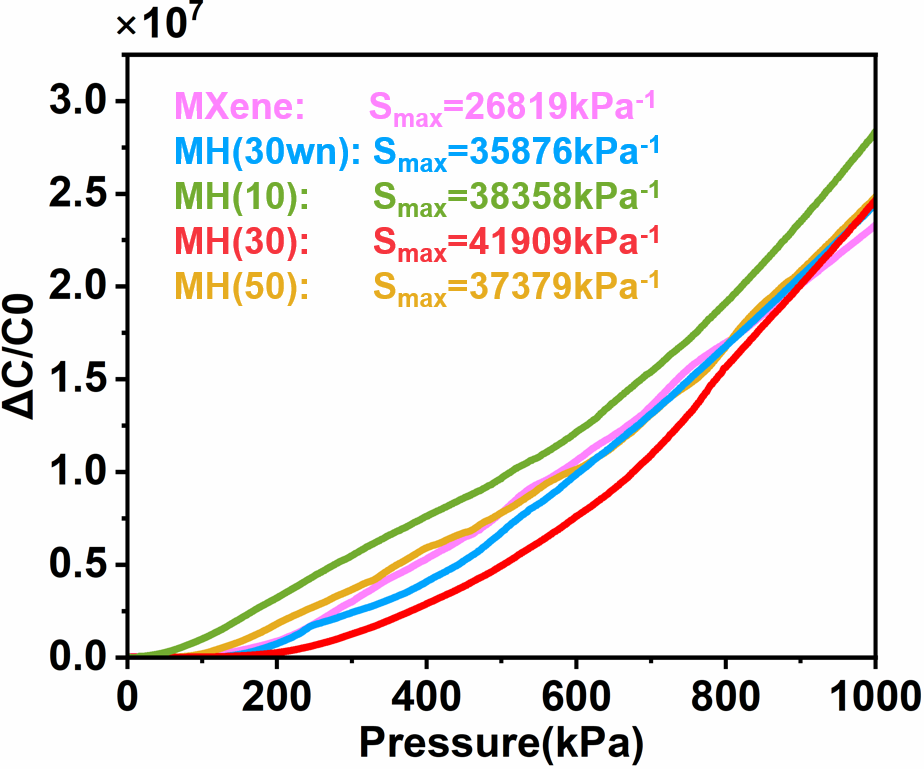


**Figure S17.** The capacitance-pressure response curves tested from different electrodes of MXene, MH(30wn), MH(10), MH(30) and MH(50).


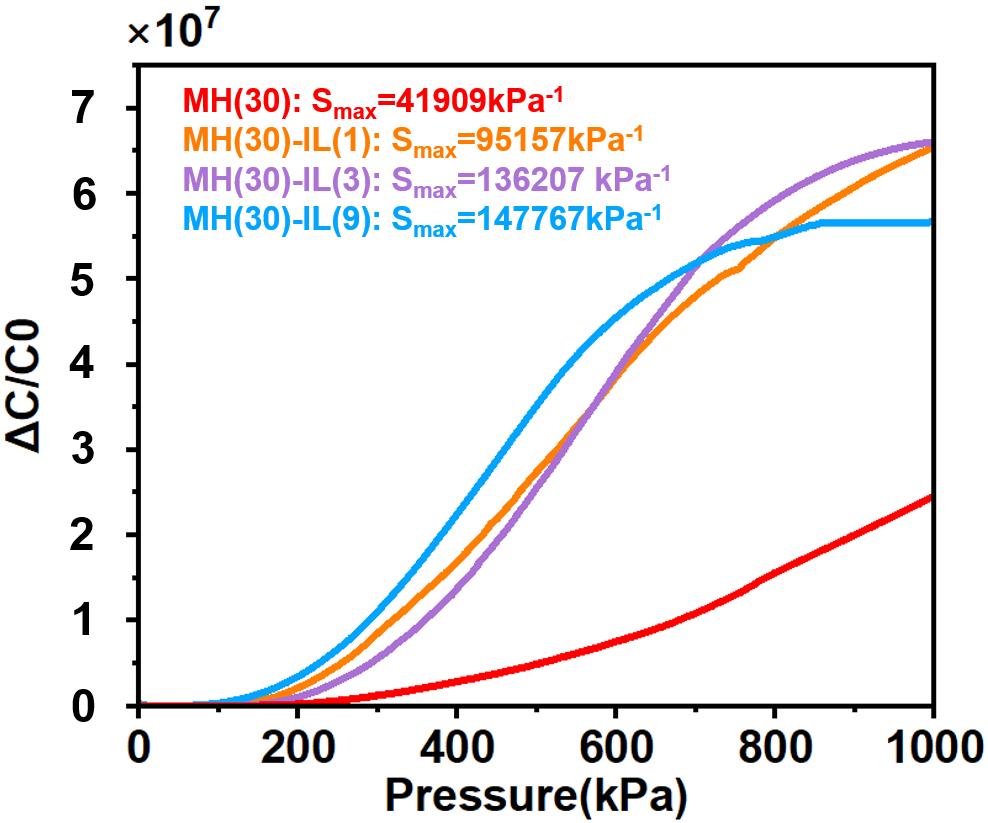


**Figure S18.** The capacitance-pressure response curves tested from different electrodes of MH(30), MH(30)-IL(1), MH(30)-IL(3) and MH(30)-IL(9).


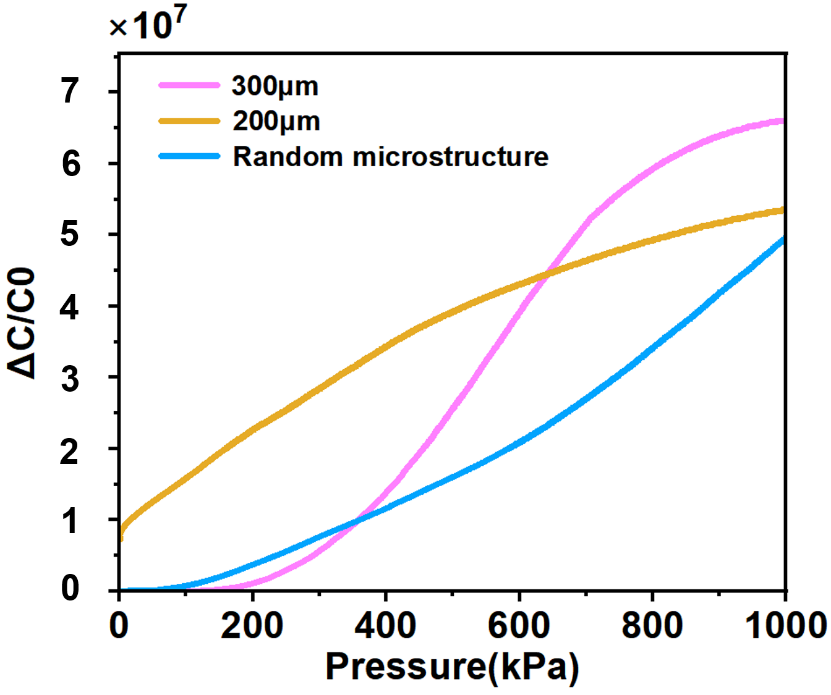


**Figure S19.** The capacitance-pressure response curves tested from different roughness of dielectric film.


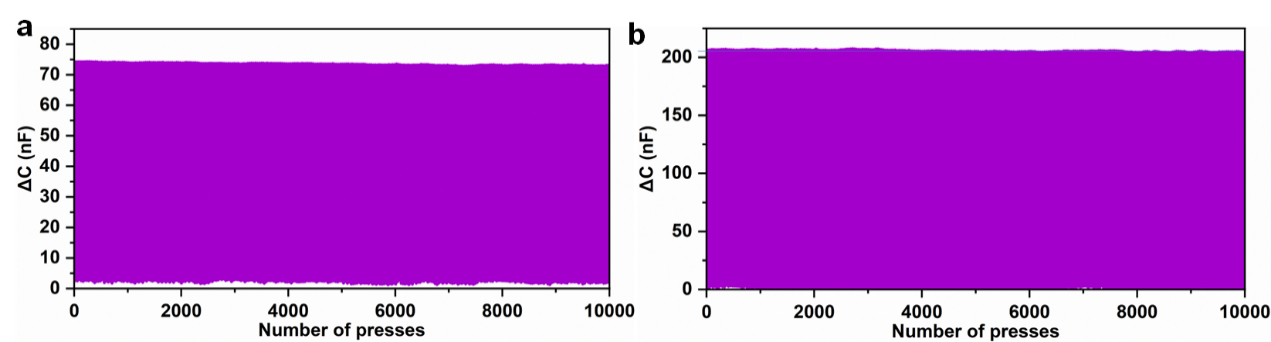


**Figure S20.** Compression-release mechanical stability over 10000 cycles under the peak pressure of 1 kPa for (a) MXene, (b) MH(30), respectively.


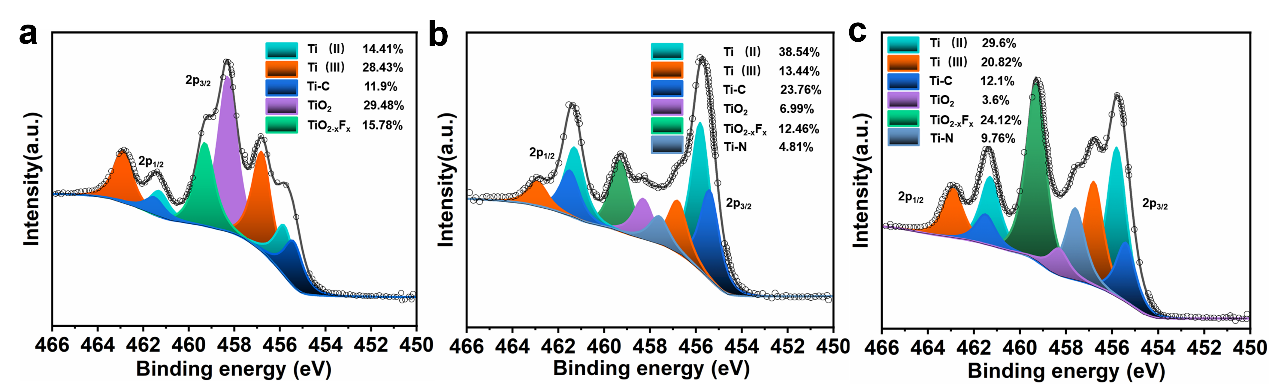


**Figure S21.** XPS spectra of Ti 2p for (a) MXene, (b) MH(30), (c) MH(30)-IL being tested after 10000 compression-release cycles.


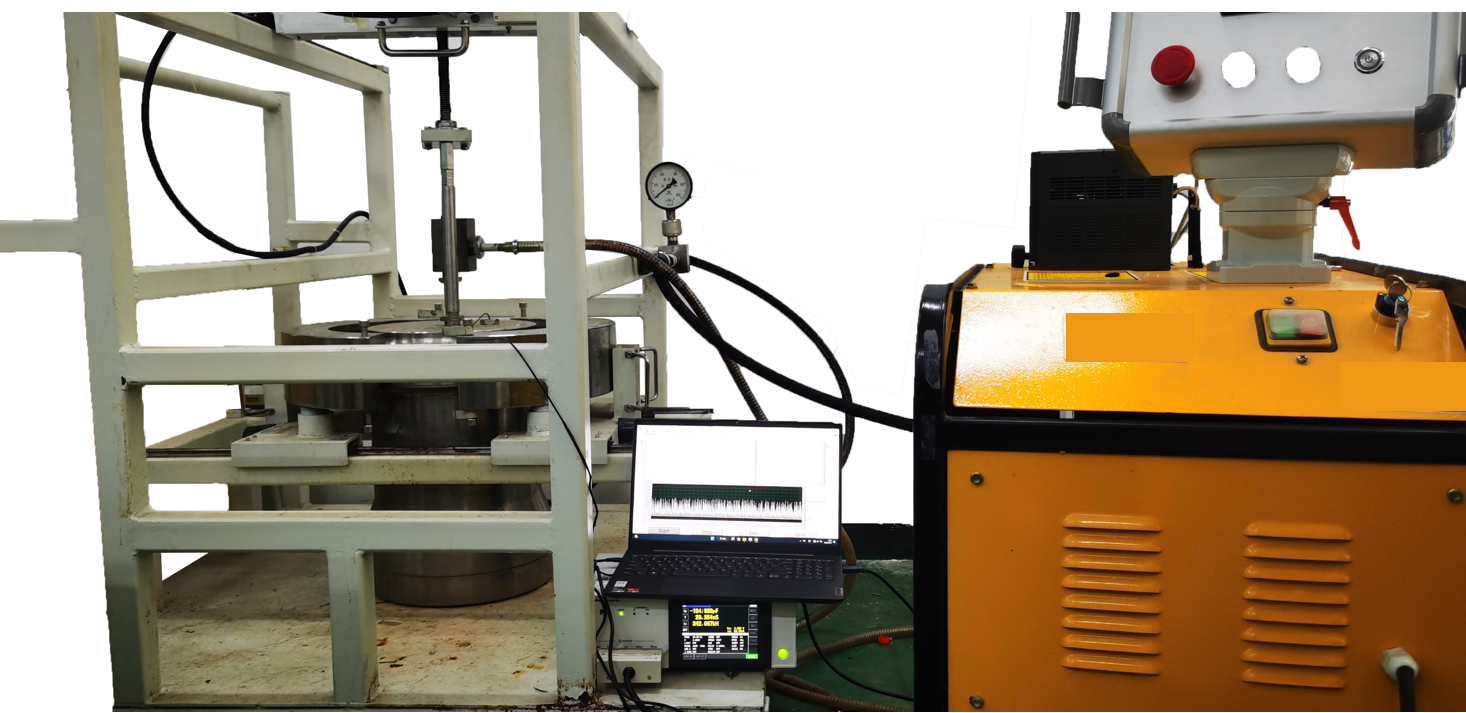


**Figure S22.** The hydraulic test system to simulate the aquatic environments with variable depths.


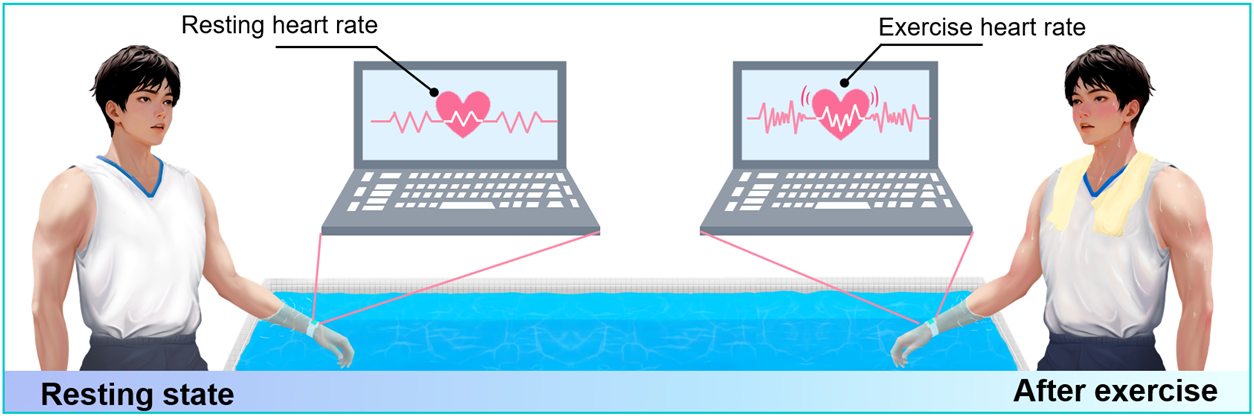


**Figure S23.** Schematic illustration of pulse waveforms monitoring in aquatic situation used by our sensing unit.


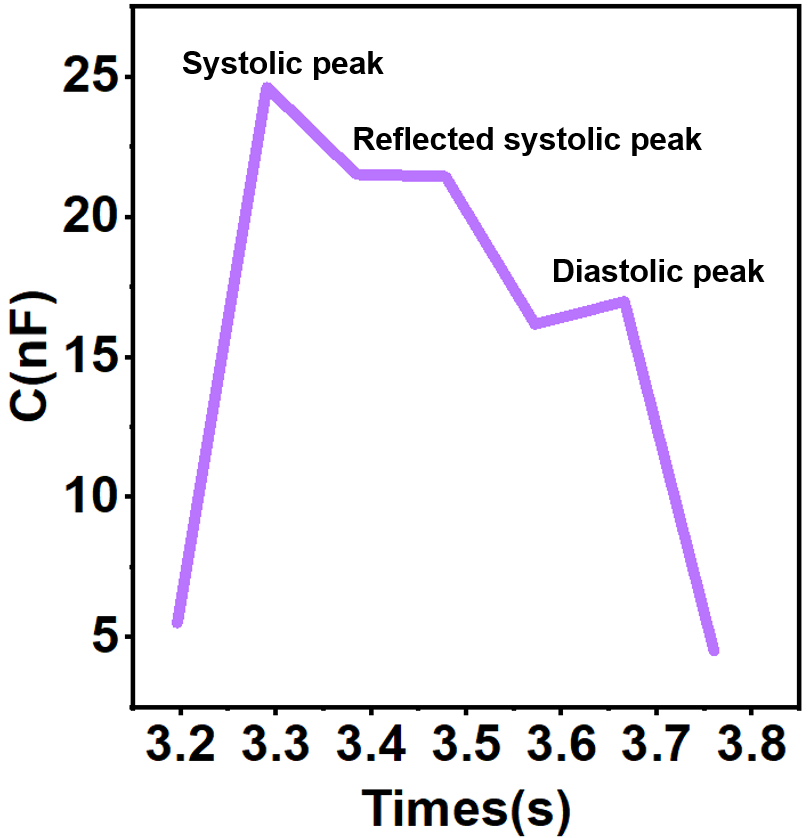


**Figure S24.** An individual cycle of pulse waveform extracted.


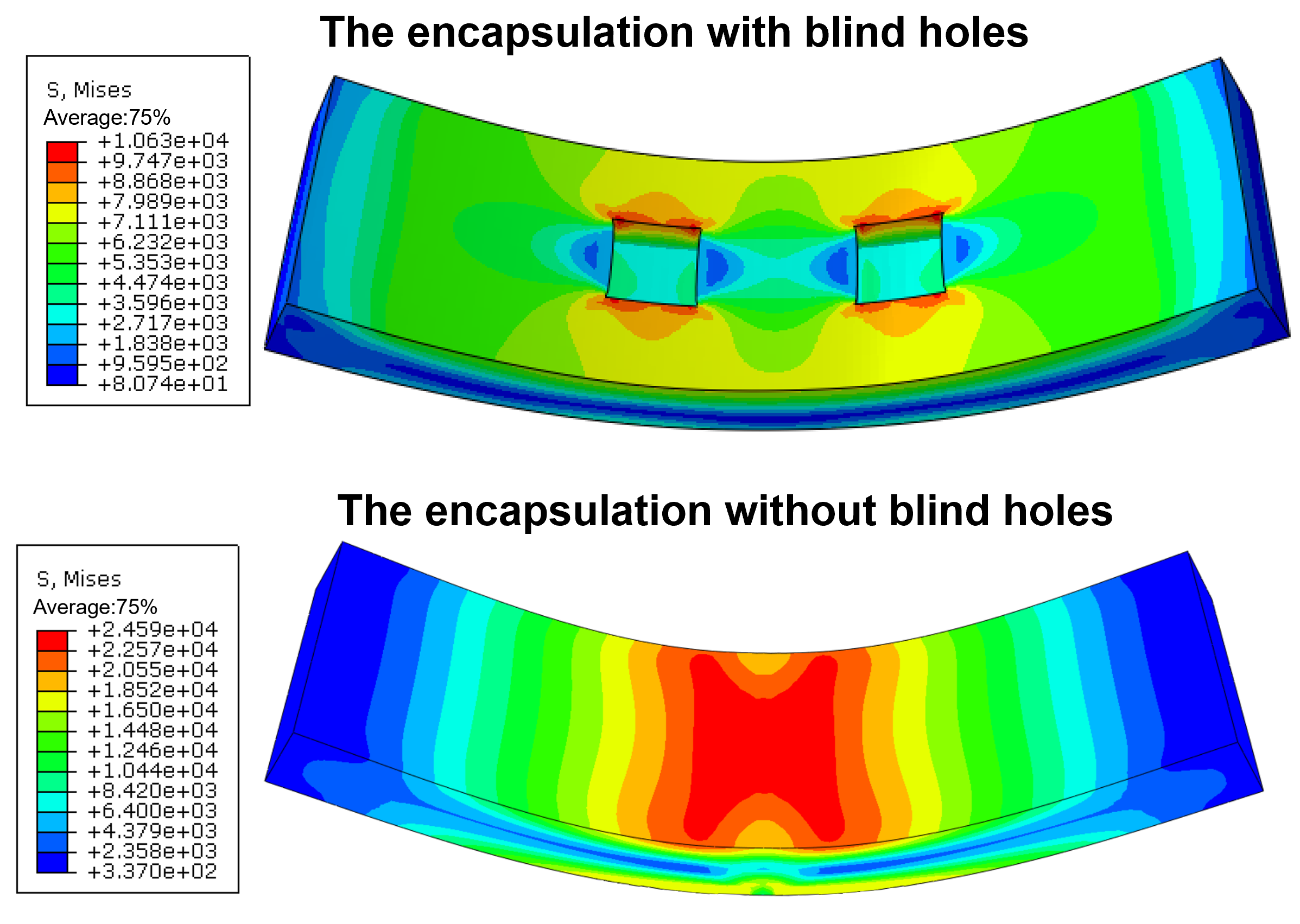


**Figure S25.** Finite element analyses of stress distribution of PDMS encapsulation with and without blind holes.


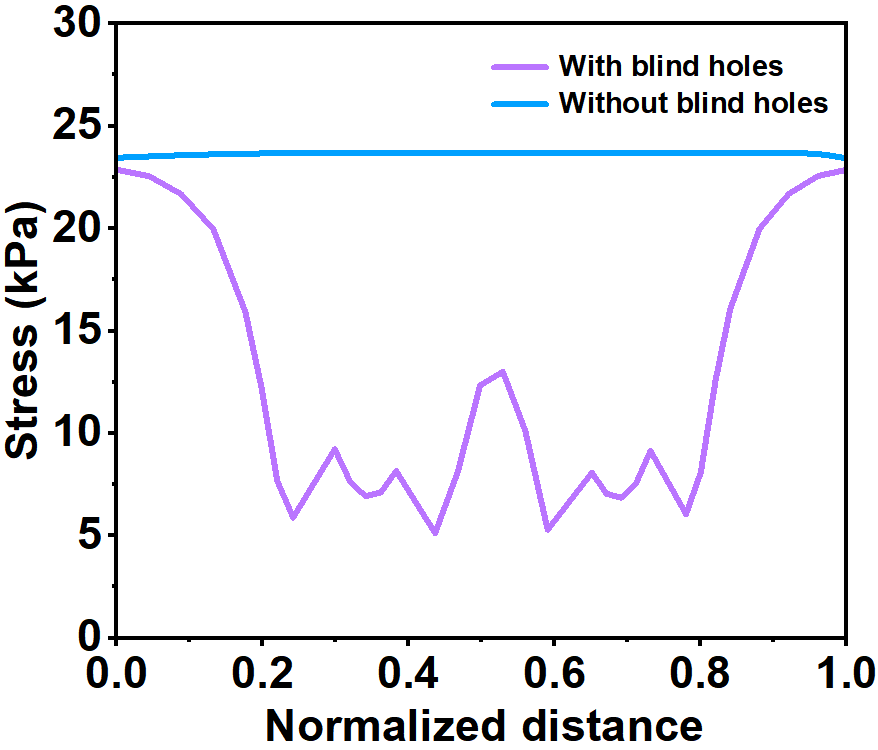


**Figure S26.** After normalizing the length of the encapsulation, we found a significant reduction in the additional stress infiltrating into the inside of two blind holes, compared to structure without blind holes.


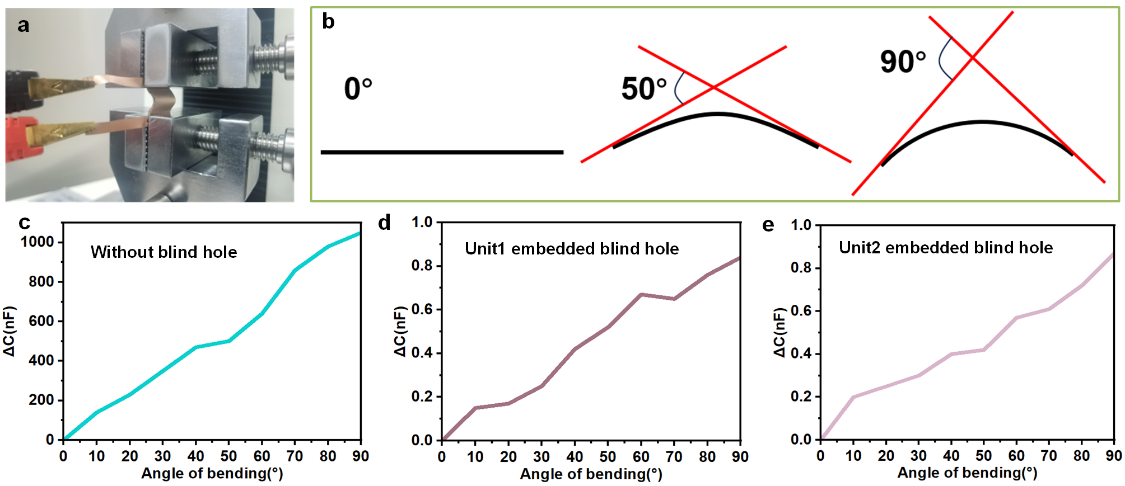


**Figure S27.** The bending tests of the sensing unit. (a) The image of sensing unit bending test. (b) The definition of bending angle. (c) The capacitance changing of sensing unit without blind hole protection. (d, e) The capacitance changings of unit1 and unit 2 embedded in blind hole, respectively.


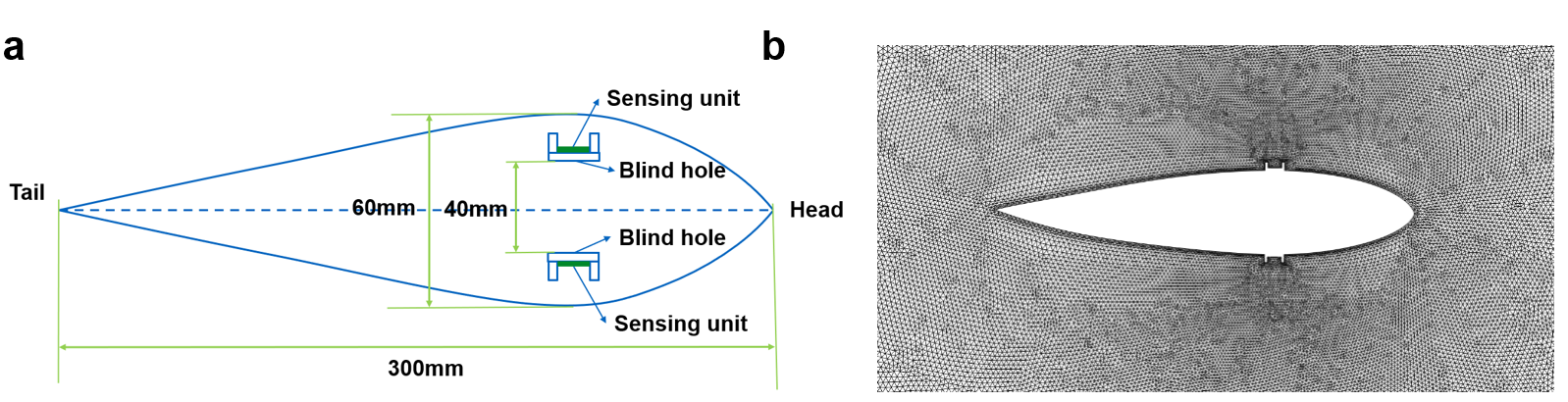


**Figure S28.** Finite element analyses of fish Swimming. (a) The size and relative position parameters of the fish and the sensing units it wears (Top view of the fish). (b) Arbitrary Lagrangian-Eulerian (ALE) of the interaction of fluid and the fish.


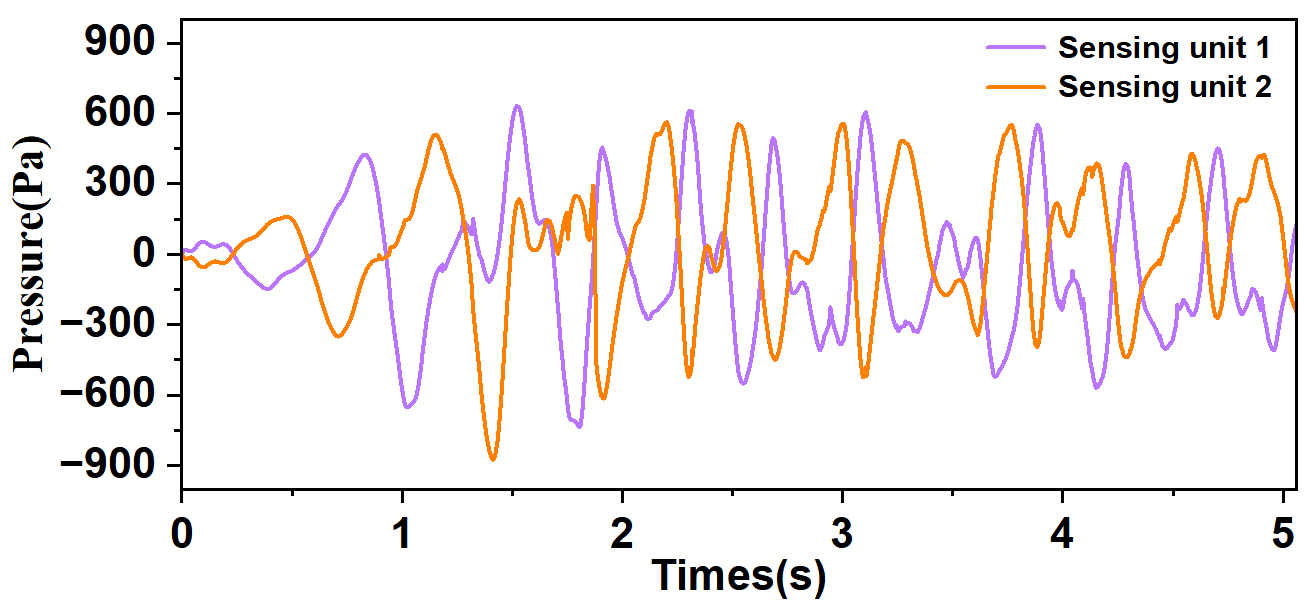


**Figure S29.** The pressure waveforms captured by the sensing units located on both symmetric sides of the fish.


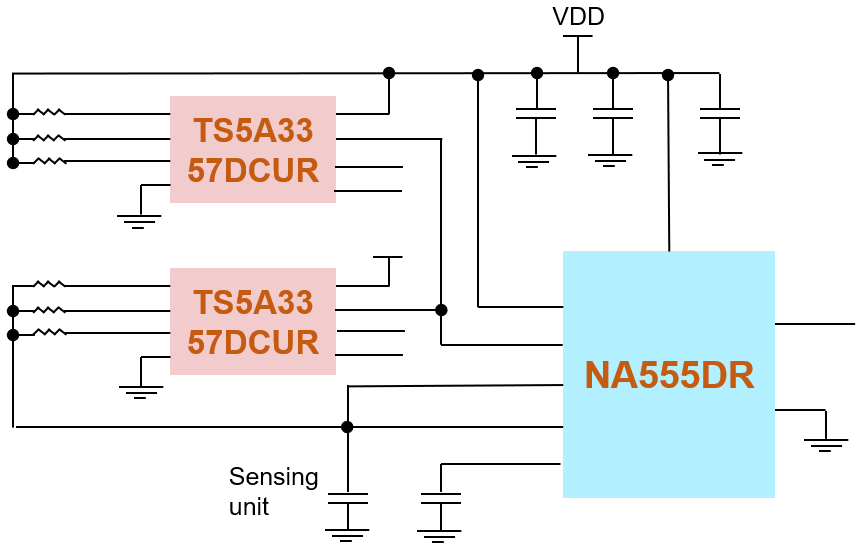


**Figure S30.** The 555 timer-based capacitance-frequency conversion (CFC) circuit


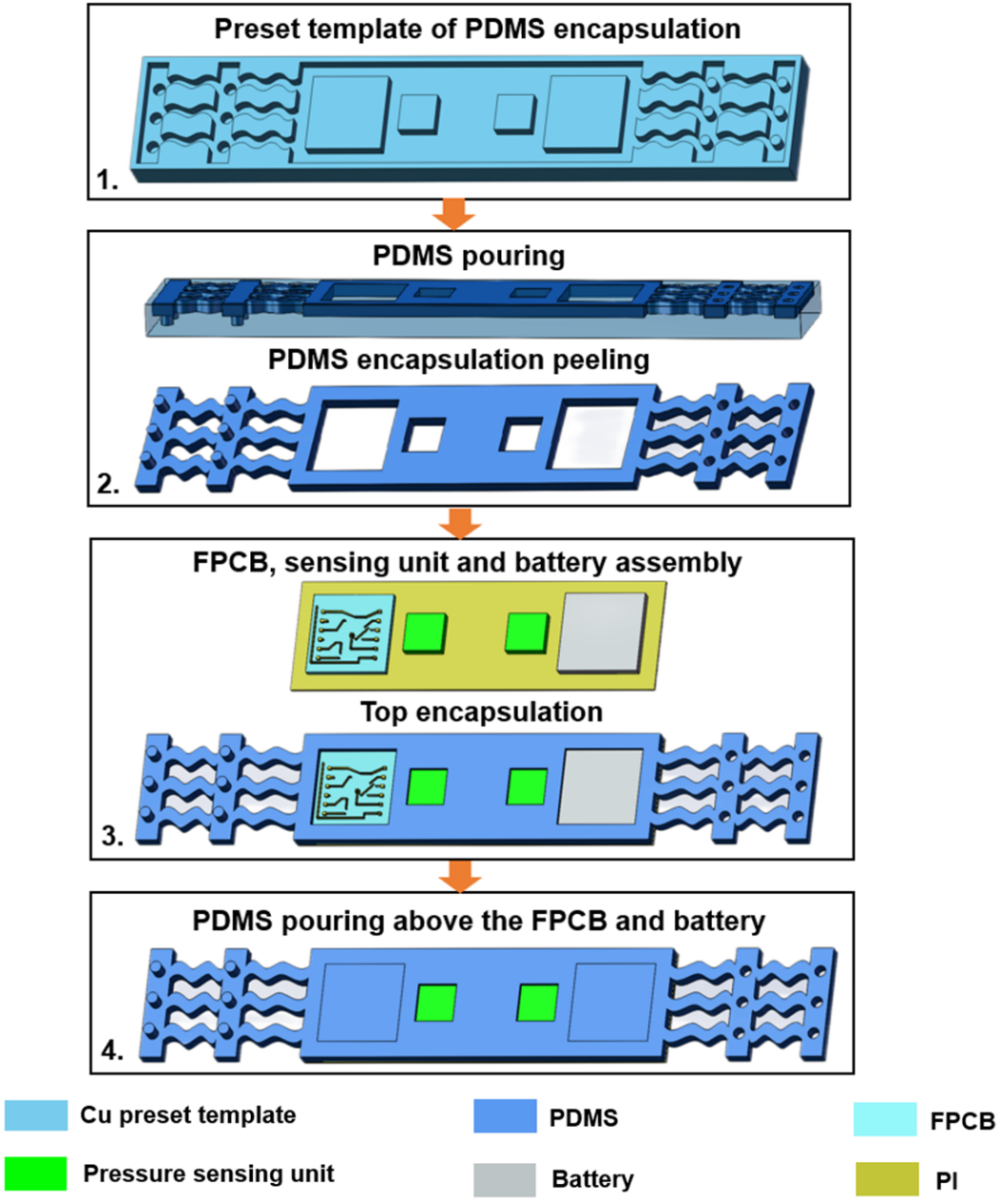


**Figure S31.** The schematic process fabrication for the underwater vest system.

**
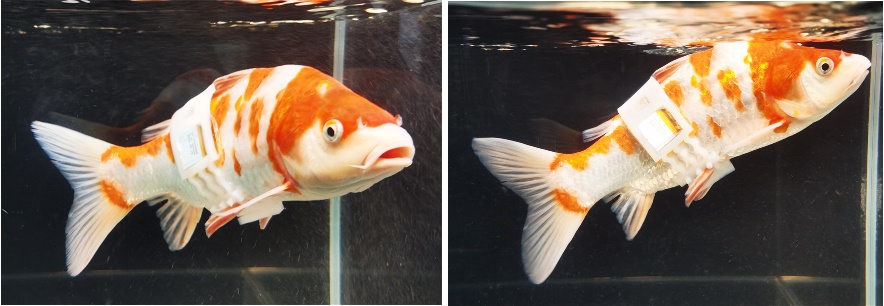
**

**Figure S32.** An underwater vest attached to koi fish.


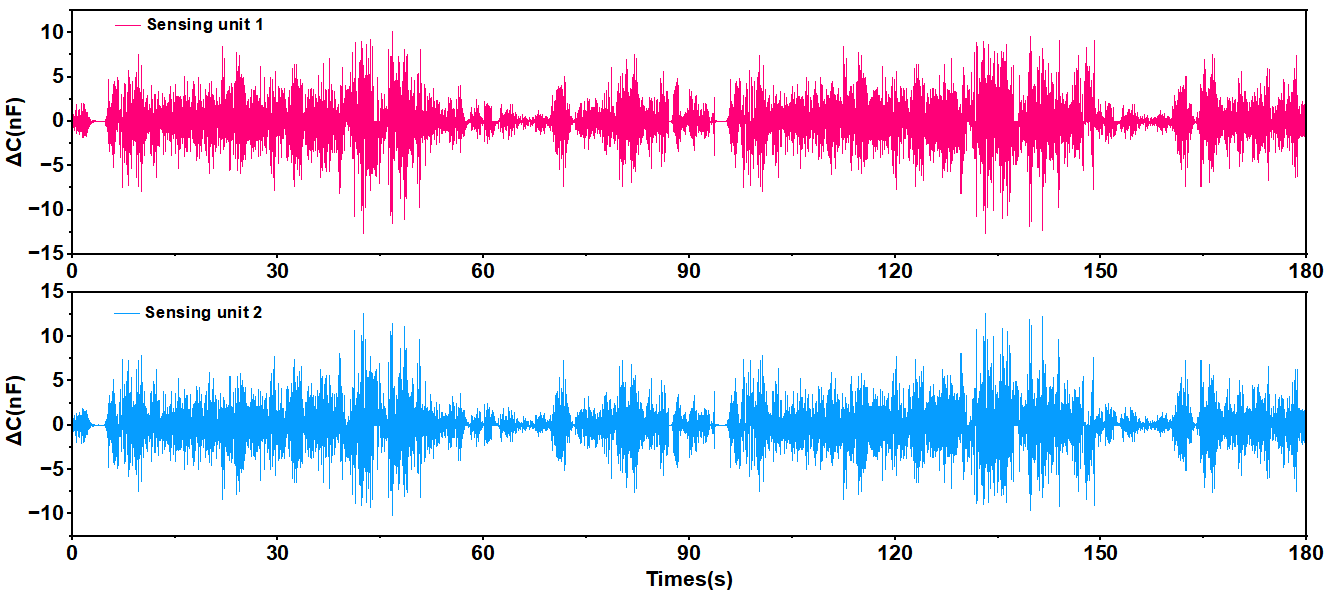


**Figure S33.** The complete capacitance outputs of a fish swam in turning left.


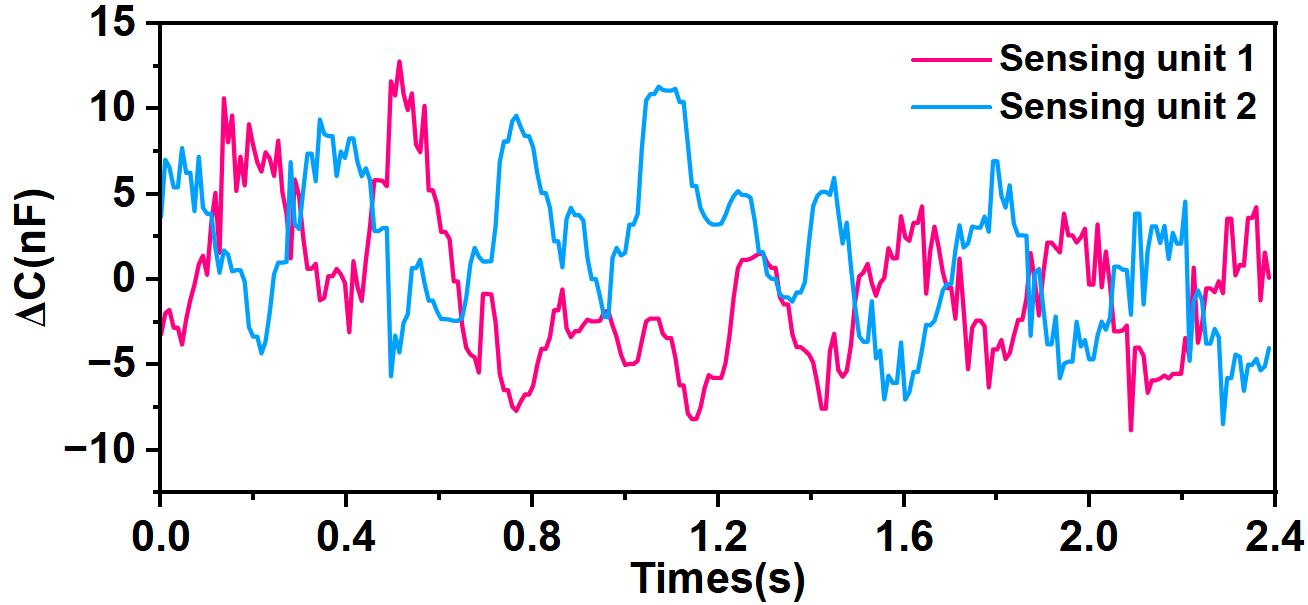


**Figure S34.** The complete capacitance outputs of a fish swam in turning left.


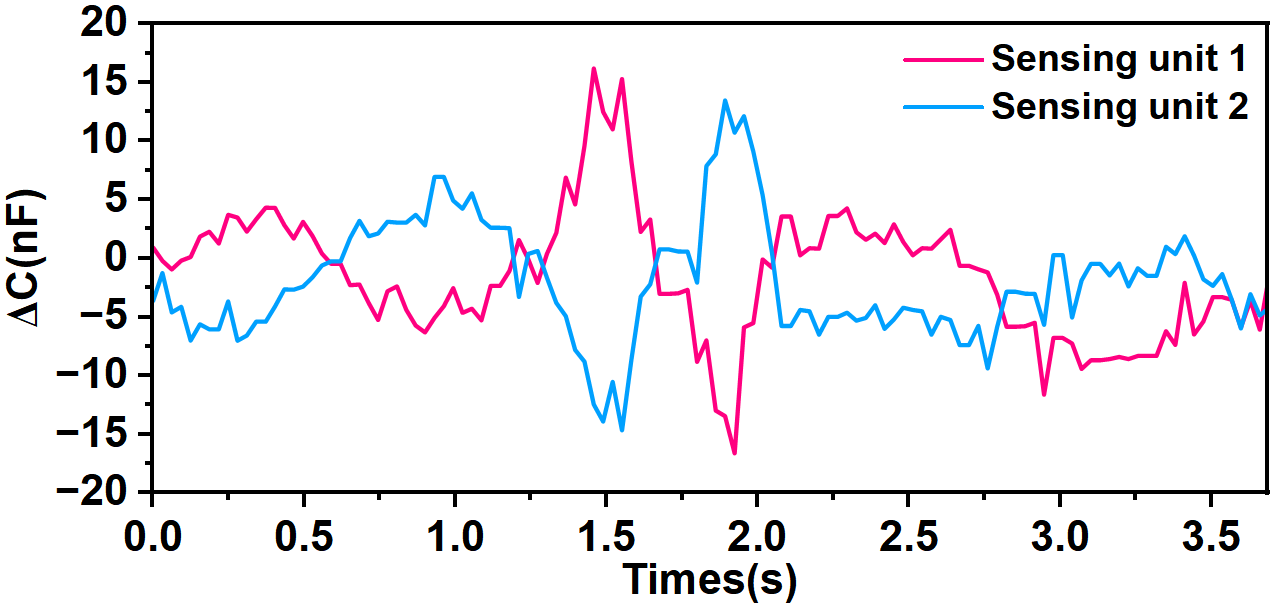


**Figure S35.** The capacitance outputs of a fish swam after being light stimulated.


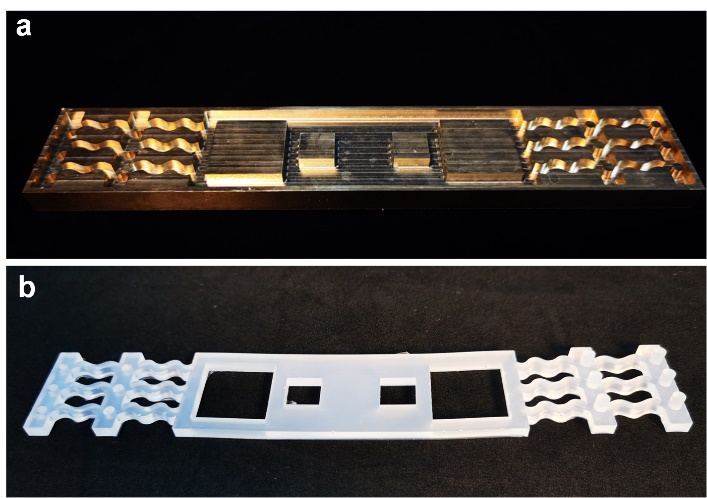


**Figure S36.** (a) Optical image of the preset template of underwater vest for pouring PDMS and curing. (b) Optical image of the PDMS encapsulation.


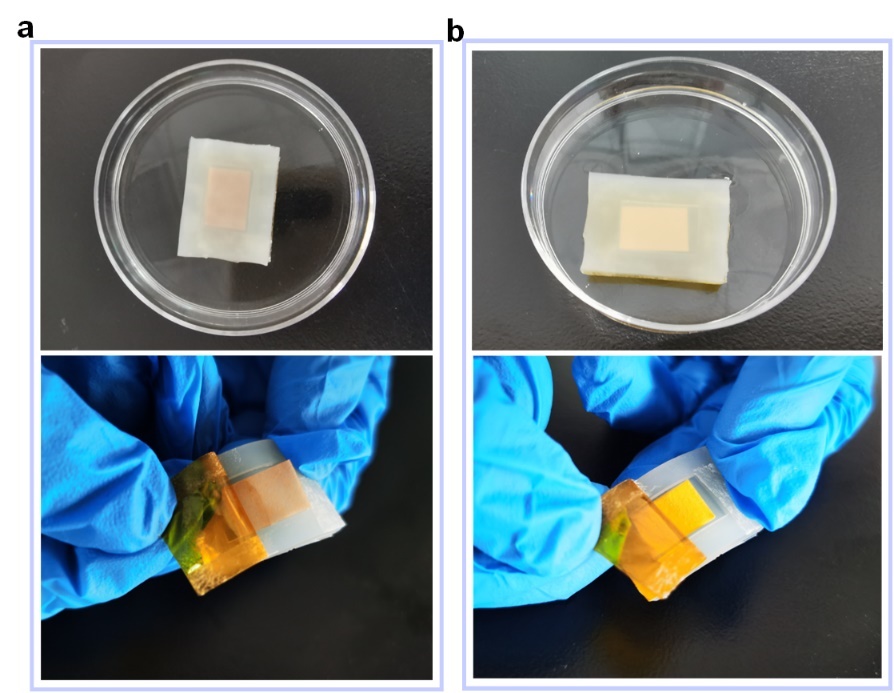


**Figure S37.** (a) The waterproof effect without O_2_ plasma treatment of PDMS encapsulation. (b) The waterproof effect with O_2_ plasma treatment of PDMS encapsulation.

**References**

1. Zhang, L. et al. Highly sensitive capacitive flexible pressure sensor based on a high-permittivity MXene nanocomposite and 3D network electrode for wearable electronics. *ACS sens.* **6**, 2630-2641 (2021).
2. Niu, H. et al. Highly morphology‐controllable and highly sensitive capacitive tactile sensor based on epidermis‐dermis‐inspired interlocked asymmetric‐nanocone arrays for detection of tiny pressure. *Small* **16**, 1904774 (2020).
3. Zhang, Y. et al. Highly stretchable and sensitive pressure sensor array based on icicle-shaped liquid metal film electrodes. *ACS Appl. Mater. Interfaces,* **12**, 27961-27970 (2020).
4. Kumaresan, Y., Ma, S., Ozioko, O., & Dahiya, R. Soft capacitive pressure sensor with enhanced sensitivity assisted by ZnO NW interlayers and airgap. *IEEE sens. J*. **22**, 3974-3982 (2022).
5. Sharma, S., Chhetry, A., Sharifuzzaman, M., Yoon, H., & Park, J. Y. Wearable capacitive pressure sensor based on MXene composite nanofibrous scaffolds for reliable human physiological signal acquisition. *ACS Appl. Mater. Interfaces* **12**, 22212-22224 (2020).
6. Ha, K. H. et al. Highly sensitive capacitive pressure sensors over a wide pressure range enabled by the hybrid responses of a highly porous nanocomposite. *Adv Mater*. **33**, 2103320 (2021).
7. Zhu, P. et al. Skin-electrode iontronic interface for mechanosensing. *Nat. Commun.* **12**, 4731 (2021).
8. Sharma, S. et al. Hydrogen-bond-triggered hybrid nanofibrous membrane-based wearable pressure sensor with ultrahigh sensitivity over a broad pressure range. *ACS nano* **15**, 4380-4393 (2021).
9. Shi, J. et al. Embedment of sensing elements for robust, highly sensitive, and cross-talk–free iontronic skins for robotics applications. *Sci. Adv.* **9**, eadf8831 (2023).
10. Li, P. et al*.* Skin-inspired large area iontronic pressure sensor with ultra-broad range and high sensitivity. *Nano Energy* **101**, (2022).
11. Lu, P. et al. Iontronic pressure sensor with high sensitivity and linear response over a wide pressure range based on soft micropillared electrodes. *Sci. Bull*. **66**, 1091-1100, (2021).
12. Bai, N. et al. Graded intrafillable architecture-based iontronic pressure sensor with ultra-broad-range high sensitivity. *Nat. Commun*. **11**, 209, (2020).
13. Liu, Q. et al*.* High-porosity foam-based iontronic pressure sensor with superhigh sensitivity of 9280 kPa^-1^. *Nanomicro. Lett.* **14**, 21, (2021).
14. Wang, P., Li, G., Yu, W., Meng, C. & Guo, S. Flexible pseudocapacitive iontronic tactile sensor based on microsphere‐decorated electrode and microporous polymer electrolyte for ultrasensitive pressure detection. *Adv. Electron. Mater.* **8**, (2022).
15. Yang, C. et al*.* In *2022 IEEE Sensors.* (IEEE, 2022), pp. 1-4.
16. Gao, L. et al*.* Highly sensitive pseudocapacitive iontronic pressure sensor with broad sensing range. *Nanomicro. Lett.* **13**, 140, (2021).
17. Popov, V. L. Contact mechanics and friction: pp. 231-253. (Berlin: Springer Berlin Heidelberg, 2010).
18. Comsol, A. B. CFD module user’s guide. (COMSOL AB, 90, 2015).
19. Comsol, A. B. Structural mechanics module user’s guide. (COMSOL Multiphysics (TM) v, 5, 2012).
